# Oleocanthal as a Multifunctional Anti-Cancer Agent: Mechanistic Insights, Advanced Delivery Strategies, and Synergies for Precision Oncology

**DOI:** 10.3390/ijms26125521

**Published:** 2025-06-09

**Authors:** Shirin Jannati, Adiba Patel, Rajashree Patnaik, Yajnavalka Banerjee

**Affiliations:** 1 Department of Basic Medical Sciences, College of Medicine, Mohammed Bin Rashid University of Medicine and Health Sciences, Dubai Health, Dubai P.O. Box 505055, United Arab Emirates; 2 Birla Institute of Technology & Science, Pilani—Dubai Campus (BITS Pilani, Dubai Campus), Dubai International Academic City, Dubai P.O. Box 345055, United Arab Emirates; f20210111@dubai.bits-pilani.ac.in

**Keywords:** oleocanthal, polyphenols, cancer metabolism, nanoparticle drug delivery, gut microbiome, precision oncology, lysosomal membrane permeabilization, Mediterranean diet

## Abstract

Oleocanthal (OC), a secoiridoid phenolic compound exclusive to extra virgin olive oil (EVOO), has emerged as a promising nutraceutical with multifaceted anti-cancer properties. Despite its well-characterized anti-inflammatory and antioxidant effects, the mechanistic breadth and translational potential of OC in oncology remain underexplored and fragmented across the literature. This comprehensive review synthesizes and critically analyzes recent advances in the molecular, pharmacological, and translational landscape of OC’s anti-cancer activities, providing an integrative framework to bridge preclinical evidence with future clinical application. We delineate the pleiotropic mechanisms by which OC modulates cancer hallmarks, including lysosomal membrane permeabilization (LMP)-mediated apoptosis, the inhibition of key oncogenic signaling pathways (c-MET/STAT3, PAR-2/TNF-α, COX-2/mPGES-1), the suppression of epithelial-to-mesenchymal transition (EMT), angiogenesis, and metabolic reprogramming. Furthermore, this review uniquely highlights the emerging role of OC in modulating drug resistance mechanisms by downregulating efflux transporters and sensitizing tumors to chemotherapy, targeted therapies, and immunotherapies. We also examine OC’s bidirectional interaction with gut microbiota, underscoring its systemic immunometabolic effects. A major unmet need addressed by this review is the lack of consolidated knowledge regarding OC’s pharmacokinetic limitations and drug–drug interaction potential in the context of polypharmacy in oncology. We provide an in-depth analysis of OC’s poor bioavailability, extensive first-pass metabolism, and pharmacogenomic interactions, and systematically compile preclinical evidence on advanced delivery platforms—including nanocarriers, microneedle systems, and peptide–drug conjugates—designed to overcome these barriers. By critically evaluating the mechanistic, pharmacological, and translational dimensions of OC, this review advances the field beyond isolated mechanistic studies and offers a strategic blueprint for its integration into precision oncology. It also identifies key research gaps and outlines the future directions necessary to transition OC from a nutraceutical of dietary interest to a viable adjunctive therapeutic agent in cancer treatment.

## 1. Background

### Roadmap

- *Section 1* *(Background)*: We begin by contextualizing the current challenges in oncology, highlighting the escalating global cancer burden, the persistent issues of drug resistance, the critical role of chronic inflammation in tumorigenesis, and the emerging promise of nutraceuticals, particularly polyphenols like Oleocanthal (OC), in addressing these complexities.
- *Section 2* *(OC: A Unique Phenolic Compound from EVOO)*: This section introduces OC, detailing its chemical identity as a secoiridoid dialdehyde, its characteristic organoleptic properties, and the unique structural features that underpin its biological activity.
- *Section 3* *(Oleocanthal in the Mediterranean Diet)*: Here, we examine OC within the broader framework of the Mediterranean Diet (MD), discussing the epidemiological evidence linking MD adherence to reduced cancer risk. This section critically analyzes the significant variability of OC content in xtra virgin olive oil (EVOO) due to factors such as olive cultivar, geographical origin, and processing methods. Furthermore, we delve into OC’s challenging pharmacokinetic profile, including its poor bioavailability, extensive first-pass metabolism involving Phase I and Phase II enzymatic processes, and the potential for clinically relevant drug–drug interactions in oncological settings. The influence of other dietary components within the MD on OC’s metabolism and function is also explored.
- *Section 4* *(Advanced Delivery Strategies for OC)*: Addressing the pharmacokinetic hurdles of OC, this section evaluates a spectrum of innovative delivery platforms. We discuss current modalities and their limitations, alongside emerging strategies such as liposomes, exosomes, microneedle systems, metal–organic frameworks (MOFs), dendrimer-based nanocarriers, and peptide–drug conjugates (PDCs), all aimed at enhancing OC’s stability, bioavailability, and tumor-targeting efficiency.
- *Section 5* *(Mechanisms Underlying the Potential Anti-Cancer Effects of Oleocanthal)*: This core section elucidates the pleiotropic anti-cancer mechanisms of OC. We detail its capacity to induce apoptosis via lysosomal membrane permeabilization (LMP), inhibit key oncogenic signaling pathways such as c-Met and STAT3, modulate the NLRP3 inflammasome, influence histone epigenetic pathways, modulate Th1/Th17 immune responses, inhibit the prostaglandin E2 synthesis pathway (COX-2/mPGES-1), and exert anti-angiogenic effects.
- *Section 6* *(Oleocanthal’s Modulation of PAR-2 Signaling and Calcium Homeostasis in Colorectal Cancer)*: Focusing on colorectal cancer (CRC), this section explores our recent findings on OC’s ability to selectively inhibit Protease-Activated Receptor 2 (PAR-2) expression and restore intracellular calcium homeostasis, thereby attenuating key inflammatory and tumorigenic processes in CRC models.
- *Section 7* *(Oleocanthal in Hereditary Cancer Predisposition)*: We explore the potential chemopreventive role of OC for individuals with hereditary cancer predispositions, such as BRCA1/2 mutation carriers and those with Lynch syndrome, by modulating inflammation and oxidative stress.
- *Section 8* *(Oleocanthal as a Modulator of Metabolic Programming and Tumor Bioenergetics)*: This section investigates OC’s emerging role in regulating cancer cell metabolism, including its potential effects on the Warburg effect, mitochondrial function, the pentose phosphate pathway (PPP), and lipid metabolism, underscoring its capacity to induce bioenergetic stress in tumor cells.
- *Section 9* *(Gut Microbiome, Cancer, and Oleocanthal)*: The bidirectional interaction between OC and the gut microbiome is discussed, focusing on how OC can modulate microbial composition—suppressing pro-carcinogenic species like Bacteroides fragilis and fostering beneficial bacteria—and how this interplay influences both gastrointestinal and systemic malignancies.
- *Section 10* *(Synergistic Effects and Combination Therapies)*: This section highlights OC’s significant potential in combination therapies, detailing its synergistic interactions with standard treatments (e.g., PARP inhibitors, taxanes, FLT3 inhibitors) across various cancers, including prostate, pancreatic, glioblastoma, and hematological malignancies, by mitigating chemoresistance and toxicity.
- *Section 11* *(Future Directions, Therapeutic Potential and Critical Research Priorities)*: We outline critical future research priorities to translate OC’s preclinical promise into clinical reality. This includes strategies for PAR-2 modulation in chondrosarcoma and CRC therapeutics, advancing clinical potential through PK-PD modeling and GMP-grade formulation development, employing advanced proteomics and AI-driven approaches for target identification and binding characterization, and addressing the current paucity of dedicated clinical trials.
- *Section 12* *(Material and Methods):* The methodological underpinnings of this review are described, detailing the literature search strategy, eligibility criteria for study selection, and the data extraction and synthesis processes employed.
- *Section 13* *(Conclusions):* Finally, we synthesize the comprehensive evidence presented, reaffirming OC’s status as a mechanistically versatile nutraceutical with considerable potential in precision oncology. We underscore the necessity of integrating advanced delivery systems and rational combination strategies to overcome existing limitations and harness OC’s full therapeutic capabilities against complex malignancies.

Despite significant advances in cancer therapy, the burden of malignancies continues to rise globally, with an estimated 28.4 million new cancer cases projected by 2040, reflecting a 47% increase from 2020 levels [1]. This alarming trend persists despite the advent of targeted therapies and immuno-oncology, largely due to drug resistance, immune evasion, and metabolic reprogramming—hallmarks that confer malignant resilience [2]. For instance, in non-small cell lung cancer (NSCLC), patients receiving EGFR inhibitors like osimertinib often relapse due to the emergence of EGFR C797S resistance mutations [3,4]. Similarly, colorectal cancers treated with anti-EGFR monoclonal antibodies (e.g., cetuximab) develop acquired resistance through MAPK reactivation or MET amplification [5,6,7,8]. Even in immunotherapy, only a subset of patients with high tumor mutational burden respond to immune checkpoint inhibitors, as immune exclusion and T-cell exhaustion limit durable outcomes [9].

Beyond genetic adaptations, tumors undergo metabolic rewiring, favoring glycolysis (Warburg effect), glutaminolysis, and fatty acid oxidation, which fuels proliferation and helps evade apoptosis [10]. For example, therapy-resistant triple-negative breast cancers show increased dependency on lipid biosynthesis and mitochondrial metabolism, creating challenges for standard chemotherapies [11,12].

In parallel, chronic inflammation has emerged as a central enabler of tumorigenesis and therapeutic resistance. Inflammatory mediators such as TNF-α, IL-6, and prostaglandins activate key oncogenic pathways—NF-κB, STAT3, and COX-2—which promote cell survival, angiogenesis, and immune suppression [13,14,15]. For example, colitis-associated CRC arises from persistent inflammatory signaling that fosters genomic instability and tumor progression [16]. Moreover, inflammation-induced epithelial–mesenchymal transition (EMT) contributes to metastasis and reduces sensitivity to both chemotherapy and targeted agents [17]. Cancer-associated fibroblasts (CAFs), driven by cytokine-rich inflammatory milieus, further remodel the extracellular matrix and shield tumor cells from cytotoxic T lymphocytes, thereby undermining immunotherapy [18]. Furthermore, one of the key inflammatory mediators contributing to drug resistance is PAR-2, a G-protein-coupled receptor activated by serine proteases such as trypsin and coagulation factors. PAR-2 signaling orchestrates a cascade of pro-tumorigenic events including inflammatory cytokine release, angiogenesis, epithelial–mesenchymal transition, and immune evasion [19]. Notably, the upregulation of PAR-2 has been shown to attenuate the cytotoxic efficacy of chemotherapeutic agents by activating STAT3 and MAPK pathways, both of which promote survival and reduce apoptosis in cancer cells [20,21,22]. In colorectal and breast cancer models, PAR-2 activation correlates with poor prognosis and therapeutic resistance, particularly in EGFR inhibitors and DNA-damaging agents [20,22,23,24]. Its expression in tumor-associated stromal cells, including cancer-associated fibroblasts, further potentiates microenvironmental resistance. These observations position PAR-2 not merely as a passive biomarker, but as an active effector of treatment failure, highlighting the need for novel agents capable of modulating this pathway. These data underscore the dual challenge posed by inflammation: it accelerates cancer evolution while simultaneously rendering malignant cells more refractory to treatment.

Given the complex interplay between inflammation, metabolic rewiring, and therapeutic resistance, there is increasing interest in nutraceuticals as adjuncts in oncology. These bioactive dietary compounds—particularly those derived from fruits, vegetables, and oils—exert pleiotropic effects on oxidative stress, immune modulation, and signaling pathways, often with low systemic toxicity. Unlike conventional chemotherapeutics that typically target a single molecular axis, nutraceuticals frequently exhibit multi-targeted activity, making them attractive candidates for modulating therapy-resistant tumors and restoring immune responsiveness [25,26].

Within the broad nutraceutical landscape, polyphenols stand out due to their well-characterized antioxidant, anti-inflammatory, and anti-proliferative properties [27]. Derived from plant-based sources such as green tea, berries, and olive oil, polyphenols have demonstrated the ability to inhibit key cancer hallmarks—including angiogenesis, EMT, and immune evasion—via modulation of NF-κB, STAT3, PI3K/AKT, and MAPK pathways. Importantly, several polyphenols have also been shown to re-sensitize tumors to chemotherapy, enhance the efficacy of immune checkpoint inhibitors, and remodel the tumor microenvironment [27,28,29]. Despite these promising attributes, translational progress has been hindered by variability in bioavailability, metabolic instability, and the lack of mechanistic integration in most reviews and preclinical studies.

Among these polyphenols, OC, a phenolic dialdehyde exclusive to EVOO, has garnered significant attention for its potent anti-cancer potential. Comparative studies have demonstrated that OC exhibits superior anti-inflammatory and cytotoxic effects compared to other olive-derived polyphenols such as hydroxytyrosol and oleuropein, owing to its unique dialdehydic structure and lysosomotropic activity [30,31]. Initially recognized for its NSAID-like COX inhibition, recent studies have uncovered OC’s unique mechanisms, including LMP, c-MET/STAT3 inhibition, mitochondrial depolarization, and modulation of the gut microbiome—all of which intersect with key resistance and inflammatory pathways. Moreover, OC exhibits selective cytotoxicity toward cancer cells, sparing non-malignant cells by exploiting lysosomal fragility and redox imbalance. In addition, OC is categorized under olive polyphenols approved by the European Food Safety Authority (EFSA), which has issued health claims related to the protection of blood lipids from oxidative stress, reinforcing its clinical relevance as a dietary bioactive compound. Despite its promise, the current literature on OC remains fragmented, often limited to single-mechanism studies or narrow focus on specific cancer types. Crucially, the challenges of pharmacokinetics, delivery optimization, and synergistic potential with existing therapies are seldom explored in a unified narrative.

This review addresses these critical gaps by offering the first comprehensive synthesis of OC’s anti-cancer mechanisms—spanning molecular signaling, metabolic regulation, advanced delivery systems, microbiota modulation, and precision oncology applications. It also examines synergistic roles with chemotherapeutic and targeted agents, explores delivery innovations including nanoparticles, exosomes, and microneedles, and evaluates OC’s impact in hereditary cancer syndromes. By contextualizing OC within the broader landscape of nutraceuticals and translational oncology, this review positions OC not merely as a dietary compound, but as a multifunctional therapeutic candidate with strong preclinical rationale and untapped clinical potential.

## 2. OC: A Unique Phenolic Compound from EVOO

OC, chemically known as decarboxymethyl ligstroside aglycone, is a naturally occurring secoiridoid dialdehyde found exclusively in freshly pressed EVOO. It is responsible for the characteristic peppery, throat-stinging sensation often used as a sensory indicator of EVOO quality. This distinct organoleptic property led to its discovery by Beauchamp and colleagues in 2005 [32], who observed a resemblance to ibuprofen-induced throat irritation and subsequently identified its non-steroidal anti-inflammatory activity through COX inhibition.

Structurally, OC is defined by its α,β-unsaturated dialdehyde motif, with the IUPAC name (E,3S)-4-formyl-3-(2-oxoethyl)hex-4-enoate, a molecular formula of C_17_H_20_O_5_, and a molecular weight of 304.34 g/mol [33]. Its structure incorporates the following:

(a) A carboxylic ester linkage at the C9 position.
(b) Two electrophilic aldehyde groups at C4 and C8, which enable Schiff base formation with nucleophilic targets, such as lysine residues.
(c) A conjugated enal system (C4–C5 double bond), allowing for redox activity and E/Z isomerization in solution.
(d) A defined (3S) stereochemistry and planar geometry across the enal group, both essential for its interaction with biological targets.

These chemical features confer high reactivity and biological specificity, particularly through covalent and non-covalent interactions with cellular proteins. For example, its selective inhibition of COX-2 exceeds that of ibuprofen at equimolar concentrations (41–57% vs. 13–18% inhibition at 25 µM) [34], highlighting the importance of its structural orientation and aldehydic functionality.

**Figure 1:**
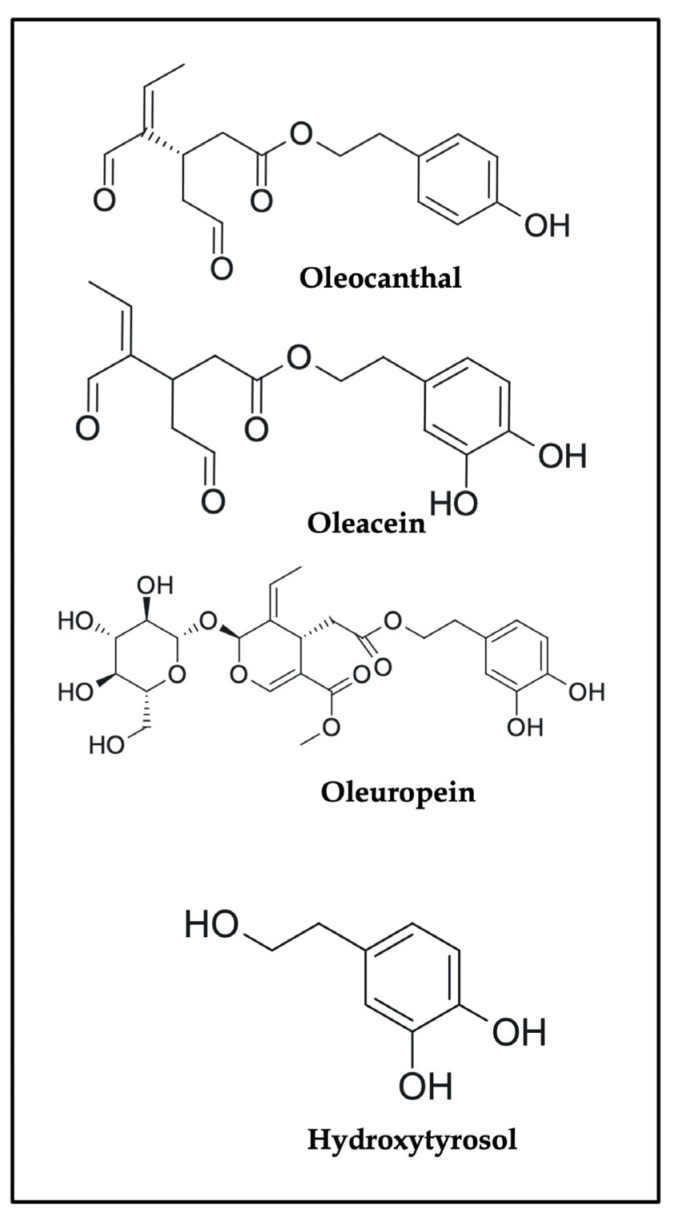
**Comparative 2D structures of key phenolic compounds in extra virgin olive oil (EVOO).** Structures of oleocanthal, oleacein, oleuropein, and hydroxytyrosol are shown to highlight variations in core scaffolds and functional groups. Oleocanthal’s unique dialdehydic secoiridoid structure distinguishes it from the other phenolics and may underlie its distinct bioactivity.

The comparative 2D structures of OC and other phenolic constituents of EVOO—namely oleacein, oleuropein, and hydroxytyrosol—are presented in Figure 1, illustrating the diversity in scaffold architecture and functional groups that contribute to their bioactivities. This visual reference highlights the structural uniqueness of OC’s secoiridoid dialdehyde core, which distinguishes its pharmacological interactions from the other olive-derived polyphenols.

While OC’s precise molecular targets and mechanisms of action are discussed in detail later in this review, its distinctive chemical architecture clearly underpins its pharmacological potential. However, realizing this potential in dietary and clinical settings requires a thorough understanding of how OC behaves in real-world consumption patterns. We now explore OC in the context of the MD, focusing on its dietary variability, absorption challenges, and metabolic considerations that influence its applicability as an anti-cancer agent.

Section Summary:

This section outlines the chemical identity of OC as a secoiridoid dialdehyde unique to EVOO, with structural features—such as electrophilic aldehyde groups and a conjugated enal system—that underpin its potent COX-2 inhibition and biological specificity. Its distinct reactivity explains its superior anti-inflammatory properties compared to other olive polyphenols. These chemical attributes form the mechanistic foundation for OC’s pharmacological promise, which is further contextualized within dietary intake and bioavailability in the next section.

## 3. Oleocanthal in the Mediterranean Diet: Bioavailability, First-Pass Metabolism, and Clinical Drug Interaction Considerations

MD is a well-established dietary pattern characterized by a high intake of vegetables, legumes, fresh fruits, non-refined cereals, nuts, and olive oil—particularly EVOO. It also includes the moderate consumption of fish and dairy, a low intake of red meats, and moderate ethanol consumption, primarily from red wine during meals. In Mediterranean countries, EVOO is a staple, consumed daily alongside main dishes such as vegetables and legumes, enhancing both flavor and nutrient bioavailability via the lipid-mediated absorption of fat-soluble polyphenols [35]. Epidemiological studies and clinical trials have consistently linked adherence to the MD with a reduced risk of various malignancies and lower cancer mortality rates, with the most robust evidence observed in breast cancer (summarized in Table 1). Given that EVOO is a fundamental component of the MD, its bioactive constituents—particularly OC—are of significant interest in elucidating the molecular mechanisms underlying the MD’s protective effects against cancer. EVOO serves as a natural carrier for OC, with epidemiological studies correlating EVOO-rich diets with reduced cancer incidence [36,37], details of which are shown in Table 1. Understanding this dietary framework helps contextualize OC’s bioavailability, absorption, and real-world impact, as dietary fat enhances polyphenol uptake by facilitating micelle formation and lymphatic transport [35]. Furthermore, linking the MD’s protective effects to molecular mechanisms of OC can bridge epidemiological findings with biochemical pathways, reinforcing its potential as a dietary-based cancer prevention strategy.

While the MD is widely recognized for its health benefits, including potential anti-cancer effects, it is important to note that OC cannot be singled out as the sole bioactive component responsible for these effects. The diet consists of various nutraceuticals, and the specific contribution of OC remains difficult to quantify due to its high variability in EVOO, ranging from <100 mg/kg to >1000 mg/kg depending on agronomic and processing factors [30,31,56]. The OC content in EVOO varies significantly (Table 2) due to several factors, including the following:

(a) Olive Cultivar: Certain cultivars, such as Coratina, exhibit substantially higher OC concentrations (78.2 µg/mL), whereas others, such as Taggiasca, contain significantly lower levels (8.3 µg/mL).
(b) Geographical Location: Italian EVOOs have been reported to contain up to 191.8 µg/mL OC, whereas EVOOs from the United States typically exhibit lower levels (~22.6 µg/mL).
(c) Agricultural Techniques: Increased irrigation reduces OC content, suggesting that water stress conditions enhance OC biosynthesis via activation of phenylpropanoid pathways.
(d) Olive Maturity and Harvest Time: OC concentration is dependent on the degree of ripeness at harvest, with early harvests yielding EVOO with higher phenolic content due to increased secoiridoid biosynthesis in unripe drupes [57].
(e) Processing Methods: EVOO extraction techniques influence OC retention, with two-phase centrifugation preserving higher OC levels compared to three-phase methods by minimizing water contact and hydrolysis.
(f) Storage Conditions and Thermal Stability: The chemical stability of OC is affected by exposure to oxygen, light, and temperature fluctuations, though OC remains relatively stable when initially present in high concentrations (>200 mg/kg), which inhibit oxidative degradation [57].

These factors therefore are responsible for the inconsistent dosing of OC. Despite extensive preclinical evaluations, clinical pharmacokinetic data on OC remain conspicuously limited. To date, no large-scale human trials have systematically quantified OC plasma concentrations, metabolite profiles, or bioavailability kinetics following dietary intake or purified OC supplementation [58]. These data gaps hinder the translational potential of OC and underscore the urgent need for well-designed, dose escalation clinical studies to define its pharmacokinetic and pharmacodynamic parameters in human subjects.

Additionally, OC undergoes significant hydrolysis in the gastrointestinal tract, with only 16–33% remaining intact after 4.5 h, as shown in rat models using LC-ESI-LTQ-Orbitrap-MS quantification [58]. While EVOO shows promise as a dietary prevention strategy, its therapeutic application is limited by the lack of precise dosing due to the factors mentioned above. These variations make it challenging to determine a standardized OC intake within the MD framework. Further research is needed to identify the optimal oil source, processing methods, and consumption guidelines to ensure consistent bioactive effects.

Even if a standardized OC content could be achieved in dietary interventions, its bioavailability and metabolism present additional challenges that must be addressed before definitive conclusions can be drawn regarding its therapeutic effects. OC’s systemic bioavailability is restricted by limited absorption in the gastrointestinal tract, compounded by its poor aqueous solubility (logP ~2.8), which limits dissolution and uptake. Once absorbed, OC undergoes extensive first-pass metabolism, reducing its systemic circulation levels. Its metabolism occurs through both Phase I (CYP450-mediated hydroxylation/hydrogenation) and Phase II (UGT-mediated glucuronidation) enzymatic processes [58]. Phase I metabolism primarily involves hydrogenation and hydroxylation reactions that modify its chemical structure, whereas Phase II metabolism includes conjugation reactions, particularly glucuronidation, which enhances its water solubility for excretion but may also influence its bioactivity. Additionally, OC interacts with circulating compounds such as amino acids, forming conjugates like OC–glycine, which may alter its biological effects [59], though further research is needed to elucidate these interactions.

OC demonstrates relatively limited intestinal absorption, as evidenced by its low effective permeability coefficient (2.23 ± 3.16 × 10^−5^ cm/s) and apparent permeability coefficient (4.12 ± 2.33 × 10^−6^ cm/s) [60]. The extent of absorption, reflected by the area under the mesenteric blood–time curve, is also lower compared to highly permeable reference compounds like levofloxacin. This suggests moderate-to-low oral bioavailability, although higher levels are expected to reach human plasma compared to rat plasma, indicating potential species differences in absorption and metabolism [61].

The amphiphilic characteristics of OC result in partitioning between oily and aqueous phases during digestion, with a tendency toward higher concentration in the aqueous phase (68.7%) due to its polar functional groups [58]. Following oral administration, OC concentration in the stomach decreases significantly over time (36% reduction at 2 h and 74% reduction at 4.5 h), while intestinal concentrations show similar but less pronounced reductions (16% at 2 h and 33% at 4.5 h). These findings suggest extensive pre-systemic metabolism [58].

At the molecular level, OC undergoes a series of Phase I metabolic transformations. These include hydrogenation reactions mediated by NADPH-dependent aldo-keto reductases (AKRs) located in the small intestine epithelium, resulting in reduced metabolites (OC + H_2_) [62]. In parallel, hydroxylation reactions are likely catalyzed by cytochrome P450 enzymes, leading to hydroxylated derivatives (OC + OH) [58]. Additionally, OC undergoes hydration reactions, forming hydrated metabolites (OC + H_2_O), which have been identified in plasma and various tissues [63]. Certain metabolites may undergo combined modifications, such as hydroxylation and hydration (OC + OH + H_2_O), further supporting the complexity of OC metabolism [58].

The distribution of these metabolites is tissue specific. The hydrogenated metabolite (OC + H_2_) exhibits maximum concentrations (C_max_) in plasma, liver, and heart at 2 h post-administration, while its levels peak in the stomach and small intestine at 1 h and in the kidney at 4.5 h [58]. In contrast, the metabolite OC + OH + H_2_O has been identified in various tissues except the stomach and small intestine, suggesting hepatic formation after initial gastrointestinal metabolism [58].

Following Phase I metabolism, OC metabolites undergo Phase II conjugation reactions, predominantly glucuronidation. Phase II metabolism results in the formation of glucuronide conjugates of hydrogenated and hydrated metabolites, including OC + H_2_ + glucuronic acid and OC + H_2_O + glucuronic acid, the latter representing one of the two main circulating metabolites [58,60,64]. These reactions are catalyzed by UDP-glucuronosyltransferases (UGTs), which transfer glucuronic acid from UDP-glucuronic acid to the hydroxyl groups of OC metabolites, enhancing water solubility and facilitating elimination [65]. While hepatic glucuronidation constitutes the primary clearance route, significant UGT activity in the kidney and intestine also contributes to extrahepatic metabolism [58,66,67].

Interestingly, no sulfate metabolites of OC have been identified in human studies [68]. This absence may be attributed to potential inhibition of sulfotransferases by OC or the preferential and higher-capacity nature of glucuronidation over sulfation, particularly at higher doses.

These findings collectively illustrate the complexity of OC’s first-pass metabolism, characterized by limited intestinal absorption, extensive pre-systemic metabolic processing, and significant conjugation reactions that contribute to its low systemic bioavailability.

A critical consideration in this context is the potential impact of drug–drug interactions on OC metabolism, particularly in oncology settings where polypharmacy is common. Table 3 indicates anti-cancer drugs and their potential interactions with OC metabolism. Both CYP450 and UGT-mediated interactions may influence OC pharmacokinetics and therapeutic outcomes. Cytochrome P450 enzymes, involved in Phase I metabolism, can be inhibited or induced by co-administered drugs, thereby affecting the hydroxylation and hydrogenation of OC. The induction of CYP enzymes could lead to the enhanced metabolism and reduced systemic availability of OC, while inhibition may increase plasma concentrations and potentially its bioactivity.

Similarly, glucuronidation, the predominant Phase II metabolic pathway for OC, is susceptible to modulation by several anti-cancer agents. The inhibition of UGT enzymes may reduce OC clearance, leading to elevated plasma levels and increased pharmacological effects. Conversely, the induction of UGT expression may accelerate metabolic clearance, potentially compromising OC’s therapeutic efficacy [68].

Several drugs commonly prescribed in cancer therapy or supportive care settings have been identified as potential modulators of OC metabolism. Tyrosine kinase inhibitors such as imatinib, hormone receptor modulators like tamoxifen, and antiepileptics including valproic acid, carbamazepine, and phenytoin are known to interact with CYP450 and UGT enzymes, thereby altering the metabolic fate of OC [69,70,71]. Corticosteroids such as dexamethasone, frequently co-administered with chemotherapy, are potent inducers of CYP3A4 and UGT enzymes and may accelerate OC metabolism [72,73,74].

Furthermore, OC has demonstrated pharmacodynamic interactions with several anti-cancer drugs, enhancing or protecting against their cytotoxic effects. Studies have shown that OC exhibits synergistic activity with paclitaxel, tamoxifen, lapatinib, doxorubicin, and dacarbazine in various cancer cell lines [75,76,77,78]. Additionally, Oleuropein, another phenolic compound found in olive oil, has been reported to reduce cyclophosphamide-induced toxicity in animal models, suggesting a potential protective role for OC as well [79].

The molecular basis of these interactions may involve OC’s inhibition of cyclooxygenase enzymes (COX-1 and COX-2), modulation of the c-MET receptor tyrosine kinase pathway, and induction of oxidative stress through reactive oxygen species (ROS) generation. These mechanisms may potentiate the efficacy of co-administered anti-cancer agents, mitigate chemotherapy-associated toxicity, or overcome drug resistance [80,81,82].

Recently, we have demonstrated that OC significantly downregulates the expression of PAR-2 at both the mRNA and protein levels in CRC cell lines [83]. This novel finding underscores the possibility that OC may not only exert direct anti-inflammatory and anti-cancer effects but may also indirectly influence drug disposition through the modulation of PAR-2-mediated pathways.

Emerging evidence indicates that PAR-2 signaling is intricately involved in regulating the expression and function of several ATP-binding cassette (ABC) transporters and solute carrier (SLC) transporters, which govern drug efflux and influx mechanisms. For instance, PAR-2 activation shows the potential to upregulate the expression of P-glycoprotein (ABCB1) [84,85], Breast Cancer Resistance Protein (BCRP/ABCG2) [86,87], and Multidrug Resistance-associated Proteins (MRPs, particularly MRP2/ABCC2 and MRP4/ABCC4) [88,89,90,91]. These transporters play pivotal roles in determining the pharmacokinetics of various chemotherapeutic agents by mediating their cellular efflux, thereby contributing to drug resistance and therapeutic failure in oncology settings.

Given that OC attenuates PAR-2 expression, it is plausible to hypothesize that it may concurrently modulate the activity or expression of these efflux transporters, potentially enhancing the intracellular retention and efficacy of anti-cancer agents. Notably, drugs such as doxorubicin, paclitaxel, vincristine, etoposide, and methotrexate are substrates of these transporters and are frequently compromised by transporter-mediated efflux mechanisms [92,93,94].

This observation opens a promising avenue for future investigation. Specifically, it would be pertinent to examine whether the OC-mediated downregulation of PAR-2 results in decreased expression or functional activity of ABC transporters, thereby augmenting the intracellular accumulation and cytotoxicity of co-administered anti-cancer drugs. Such studies could include detailed pharmacokinetic profiling, transporter expression analysis, and functional efflux assays in relevant cancer models. Furthermore, exploring the impact of OC on the SLC family transporters—which facilitates drug uptake—would provide a comprehensive understanding of its role in modulating drug disposition and therapeutic outcomes.

Beyond OC’s intrinsic bioavailability challenges and issues associated with drug–drug interactions, its interaction with other dietary components in the MD may further modulate its metabolism and function. In fact, interplay between OC and MD components extends beyond simple conjugation reactions, involving dynamic competition between endogenous and dietary nucleophiles for OC’s electrophilic α,β-unsaturated aldehydes. Recent mechanistic studies demonstrate OC’s pH-dependent reactivity profile, with accelerated adduct formation occurring in the acidic gastric environment (pH 3.5) compared to intestinal conditions (pH 7), potentially directing metabolite formation pathways through compartment-specific reactions [59]. This pH-mediated selectivity explains the preferential formation of oleocysteine derivatives in the stomach versus the intestinal generation of oleoglutathione conjugates, creating distinct metabolite pools with differential absorption kinetics and tissue distribution patterns. Competitive binding assays reveal that sulfur-containing amino acids (cysteine, methionine) exhibit 3–5× higher reaction rates with OC compared to neutral or aromatic residues [95], suggesting MD components like garlic (rich in cysteine derivatives) may profoundly influence OC’s metabolic fate.

The MD’s characteristic combination of wine polyphenols and OC creates a complex reaction milieu where proline-rich wine peptides compete with plasma amino acids for OC conjugation. Kinetic studies using model digestive systems show wine-derived tripeptides (e.g., γ-glutamyl-cysteinyl-glycine) form stable OC adducts 40% faster than free amino acids [59], potentially redirecting OC from hepatic glucuronidation pathways toward novel metabolite production. Notably, oleoserine derivatives demonstrate enhanced blood–brain barrier permeability compared to native OC in ex vivo models [58], suggesting dietary modulation could optimize OC’s neuroprotective effects while altering systemic exposure levels.

Emerging evidence indicates MD-derived lipids may competitively bind OC’s aldehyde groups through Schiff base formation, creating transient complexes that delay gastric absorption but enhance intestinal lymphatic uptake. In simulated digestion models, co-administration with omega-3 fatty acids (abundant in MD fish sources) increases OC’s micellar incorporation by 60% while reducing free aldehyde availability for amino acid conjugation [30]. This lipid-mediated shielding effect preserves OC’s redox-sensitive phenolic groups during gastric transit, potentially explaining observed synergies between olive oil and fish consumption in Mediterranean populations [95].

Advanced delivery systems specifically address OC’s multi-compartmental metabolic challenges through molecular encapsulation strategies. Novel phytosome formulations incorporating phosphatidylcholine and cholesterol derivatives demonstrate a 90% protection of OC’s reactive aldehydes during simulated digestion, while achieving 3× higher hepatic accumulation compared to free OC in preclinical models [30]. Phase inversion temperature-engineered nanoemulsions show particular promise for intestinal-targeted release, leveraging bile salt interactions to preferentially deliver OC conjugates to enterocyte absorption pathways over passive diffusion mechanisms [58].

Section Summary:

This section situates OC within the MD, highlighting its contribution to the MD’s cancer-protective effects and addressing the wide variability in OC content due to olive cultivar, geography, harvest timing, and processing methods. Although EVOO serves as a natural delivery matrix, OC exhibits limited systemic bioavailability owing to poor aqueous solubility, extensive pre-systemic metabolism, and complex Phase I/II biotransformation, including glucuronidation and conjugation with dietary nucleophiles. The section also delineates how MD components—such as sulfur-rich amino acids, wine-derived peptides, and omega-3 lipids—influence OC’s metabolic fate, absorption kinetics, and pharmacodynamic potential. Notably, these interactions may alter the formation of bioactive conjugates, impact transporter-mediated drug disposition, and contribute to interindividual variability in therapeutic outcomes. Finally, emerging delivery technologies such as phytosomes and nanoemulsions are introduced as potential solutions to optimize OC’s stability, absorption, and tissue targeting.

## 4. Advanced Delivery Strategies for OC: Enhancing Mechanistic Precision in Cancer Therapy

While MD provides a natural delivery matrix for OC, its clinical translation faces significant pharmacological hurdles including poor bioavailability, rapid metabolism, and inadequate tumor targeting details of which are noted in Table 4. These limitations necessitate the development of innovative delivery strategies designed to overcome OC’s metabolic vulnerabilities while enhancing its mechanistic precision in cancer therapy. The key strategies are summarized in Table 5.

### 4.1. Current Delivery Modalities and Their Limitations

The direct administration of OC, either orally or intravenously, has shown promise in preclinical models, with a daily oral administration of 10 mg/kg suppressing CRC progression in mouse xenografts by 72.5% and reducing post-surgical recurrence [103]. However, OC exhibits poor pharmacokinetic properties, including rapid conjugation with glycine in biological fluids to form pharmacologically inactive oleoglycine within minutes at physiological glycine concentrations (≥300 μM) [59]. While OC can cross the blood–brain barrier in vitro [59,104,105], its instability in systemic circulation limits brain tumor applications. Furthermore, oral administration leads to extensive hepatic metabolism, evidenced by undetectable plasma levels in rats fed OC-rich olive oil, rendering direct administration impractical for therapeutic applications [58].

Indeed, liposomal encapsulation represents a rational approach to enhance OC’s stability and enable controlled release [96]. Studies using hydrogenated soya phosphatidylcholine (HSPC) liposomes have reported encapsulation efficiencies of 60–80%, varying with vesicle size. However, suboptimal release kinetics remain problematic, with OC and oleacein exhibiting slower release (≤40% in 24 h) compared to hydroxytyrosol (≥70%) in simulated body fluid, following Fickian diffusion patterns [96]. Storage instability, characterized by increasing liposome size and polydispersity index during storage, raises concerns about aggregation. While liposomes protect OC from degradation, their limited release efficiency and physical instability necessitate formulation improvements [106].

To circumvent the above issues, polymer-based nanoparticles, though not yet extensively tested for OC specifically, have shown promise for analogous compounds. For instance, pH-sensitive core–shell nanoparticles loaded with cisplatin reduced detoxification by GSH and enhanced cytotoxicity in breast cancer cells [107]. These systems could be adapted for OC delivery by targeting lysosomes—leveraging cancer cells’ enlarged lysosomes, a vulnerability highlighted in OC’s LMP (refer below for details) mechanism—and incorporating hydrophobic polymer layers (e.g., polycaprolactone) to shield drugs from cytoplasmic thiols. However, customizing polymer nanoparticles for OC’s amphiphilic nature remains largely unexplored.

### 4.2. Emerging Delivery Modalities and Strategic Innovations

Exosome-mediated targeted delivery represents a revolutionary approach for OC administration. These naturally derived extracellular vesicles (30–150 nm) offer biocompatibility, low immunogenicity, and inherent targeting capabilities [98,108,109]. Dendritic cell-derived exosomes modified with tumor-specific neoantigens have demonstrated enhanced uptake in cancer cells, a strategy adaptable to OC delivery [110]. Exosomes’ ability to cross the BBB makes them particularly promising for glioblastoma therapy, where OC’s STAT3 inhibition (refer below for details) could be maximized. While exosomes bypass hepatic first-pass metabolism and reduce systemic toxicity [97], challenges remain in scalable production under Good Manufacturing Practice (GMP) standards and achieving high drug-loading efficiency.

Another approach that can be suitably adopted is the microneedle-assisted transdermal delivery system. This approach physically breaches the stratum corneum, enabling localized or systemic delivery of OC-loaded nanoparticles [111]. Hollow microneedles combined with pH-sensitive poly(β-amino ester) nanoparticles [112] could facilitate OC release specifically in the acidic tumor microenvironment. This approach may be promising for melanoma treatment, where OC’s anti-inflammatory properties could not only mitigate metastasis but also deliver therapeutic agents in a localized, efficient, and minimally invasive manner. Recent studies underscore the translational relevance of microneedle-mediated OC delivery in melanoma therapy. Notably, a 2024 preclinical investigation demonstrated that dissolvable hyaluronic acid microneedle patches co-loaded with polyphenols and photothermal agents achieved localized melanoma suppression without systemic toxicity [100]. Furthermore, a 2023 meta-analysis highlighted the superior transdermal penetration efficiency and patient compliance of microneedle platforms in melanoma management compared to conventional topical formulations [99]. These advances validate the feasibility of adapting similar microneedle systems for targeted OC administration in cutaneous malignancies. Traditional melanoma treatments, such as surgical resection, chemotherapy, radiotherapy, and photodynamic therapy (PDT), face several limitations, including high recurrence rates, systemic toxicity, and limited penetration depth of therapeutic agents. Microneedles, as a transdermal drug delivery system, offer a novel approach to overcoming these challenges by ensuring precise and controlled drug administration directly into the tumor microenvironment [99]. Furthermore, microneedle array patches (MAPs) allow self-administration and sustain drug release over days, though ensuring mechanical stability during skin penetration requires polymer optimization [100], with curcumin-based systems demonstrating efficacy in melanoma treatment. This success holds particular relevance for OC due to the compounds’ shared structural and functional properties. Both curcumin and OC are polyphenolic molecules with similar hydrophobicity, molecular weights, and bioactive profiles. These parallels suggest that microneedle platforms optimized for curcumin could serve as a blueprint for OC delivery. In preclinical studies, dissolvable hyaluronic acid (HA) microneedles co-loaded with curcumin nanodrugs and photothermal agents like IR820 have achieved localized tumor regression through chemo-photothermal synergy. Such systems address critical challenges common to both compounds: poor oral bioavailability, systemic toxicity, and the need for tumor-specific accumulation. The microneedle platform’s ability to concentrate hydrophobic agents directly in the tumor microenvironment, coupled with rapid payload release upon dissolution, offers a viable strategy to overcome OC’s pharmacokinetic limitations. Furthermore, curcumin’s combinatorial potential with adjuvants (e.g., IR820) [113] underscores OC’s untested but plausible synergies in similar formulations. Animal studies further confirmed these findings, showing substantial tumor growth inhibition in microneedle-treated groups compared to controls. The localized hyperthermia not only ablated tumor tissues but also prevented metastasis, as evidenced by a reduction in lung metastases in treated animals. Moreover, histological analyses of major organs indicated that microneedle-based therapy did not cause significant systemic toxicity, highlighting its safety and specificity. Future studies should prioritize the OC-specific optimization of microneedle parameters, including drug stability, release kinetics, and synergy with adjunctive therapies, building upon the existing curcumin framework to accelerate translational potential.

Metal–organic frameworks (MOFs), such as ZIF-8, offer exceptional OC loading capacities (~50% *w*/*w*) due to their ultra-high porosity (surface area > 1000 m^2^/g) and tunable pore sizes [101,114]. Functionalization with folic acid (FA) or glucose ligands enhances tumor targeting, with FA-conjugated ZIF-8/OC composites demonstrating 80% uptake in folate receptor-positive breast cancer cells [101]. MOFs enable stimuli-responsive release; for example, glucose-coated MOFs disassemble in the high-glucose tumor microenvironment (TME), releasing OC selectively [115]. While MOFs effectively protect OC from glycine conjugation in plasma, their biodegradation kinetics in vivo require further validation.

Dendrimer-based multifunctional carriers, particularly polyamidoamine (PAMAM) dendrimers with their hyperbranched structure, allow precise surface modifications for dual targeting and imaging [102]. G4 PAMAM dendrimers conjugated to N-acetylgalactosamine (GalNAc) can target hepatocytes via the asialoglycoprotein receptor (ASGPR), achieving >90% hepatic uptake. Co-loading OC and siRNA against c-MET could enhance apoptosis in hepatocellular carcinoma, a strategy similar to that which has been used for inclisiran [116]. Dendrimers penetrate dense tumor stroma via the enhanced permeability and retention (EPR) effect, though cationic dendrimers require PEGylation to mitigate cytotoxicity.

Peptide–drug conjugates (PDCs) offer a targeted strategy to enhance the delivery and efficacy of bioactive compounds like OC in cancer therapy. These conjugates consist of three key components: a tumor-homing peptide, a biodegradable linker, and a cytotoxic payload; in this case, OC. The peptide directs the conjugate to receptors overexpressed in tumors, such as carbonic anhydrase IX (CAIX) or VEGFR in renal cell carcinoma (RCC), while the linker ensures stable circulation and controlled drug release within the tumor microenvironment. For renal tumors, OC’s therapeutic potential could be amplified through conjugation to peptides targeting CAIX or integrins, which are highly expressed in RCC. This approach would localize OC to malignant cells, mitigating its off-target effects. Critical to this design is the linker chemistry: enzyme-cleavable (e.g., cathepsin B-sensitive), acid-sensitive, or reducible disulfide linkers can exploit RCC’s unique microenvironment to trigger OC release. Self-immolative spacers further refine this process, ensuring OC remains inert until reaching the tumor. By combining OC’s inherent anti-cancer properties with PDC technology and leveraging receptor-specific targeting, this strategy could achieve precise, potent renal tumor therapy with minimal systemic toxicity [117]. The integration of tumor-targeting linkers with OC delivery offers a highly personalized medicine approach, enhancing drug efficacy while reducing side effects. Advances in linker chemistry, particularly in enzyme-cleavable, pH-sensitive, and bioorthogonal strategies, provide a solid foundation for developing next-generation OC drug conjugates tailored for renal tumor treatment.

Scorpion venom peptides, such as chlorotoxin, offer a promising platform for targeted cancer therapy due to their small size, stability, and ability to evade immune detection. These peptides can be engineered to deliver bioactive compounds like OC directly to tumors, leveraging their natural affinity for certain overexpressed cancer cell targets. Unlike conventional chemotherapeutics, OC’s anti-cancer properties—including anti-inflammatory, pro-apoptotic, and anti-angiogenic effects—could be amplified while minimizing off-target toxicity through conjugation to venom-derived peptides. Chlorotoxin’s specificity for glioma cells demonstrates the potential for tailoring venom peptides to other malignancies, such as renal or breast cancers. By modifying the peptide scaffold—for instance, introducing metal-binding residues or tumor-specific targeting sequences—researchers could create OC-peptide conjugates that exploit TME features (e.g., low pH, enzymatic activity) for controlled drug release. Notably, chlorotoxin’s blood–brain barrier penetration could further enhance OC’s delivery to otherwise inaccessible brain tumors. The inherent stability of scorpion venom peptides, conferred by disulfide bonds, ensures that OC remains protected during systemic circulation. Coupling OC to these peptides via cleavable linkers would enable tumor-specific activation, combining OC’s mechanistic potency with precision targeting. This approach could address OC’s pharmacokinetic challenges while capitalizing on its multi-targeted anti-cancer activity [118,119].

Self-assembled nanoparticles (SANPs) with aggregation-induced emission (AIE) properties offer another innovative approach. Amphiphilic OC derivatives can self-assemble into micelles (10–50 nm) under physiological conditions, enabling real-time tracking. SANPs functionalized with tumor-penetrating peptides (e.g., iRGD) enhance permeability in glioblastoma models and bypass reticuloendothelial system (RES) clearance due to their small size. However, controlling self-assembly kinetics to prevent premature drug release remains challenging.

Three-dimensionally printed personalized nutraceutical implants represent an emerging strategy for localized OC delivery. Biodegradable scaffolds loaded with OC-polymer composites (e.g., PLGA or chitosan) could provide sustained release post-resection for peritoneal cancers. While patient-specific dosing and geometry improve therapeutic precision, regulatory hurdles for customized medical devices must be addressed.

Ligand-targeted delivery systems hold significant promise for OC. Hepatocyte targeting can be achieved using nanoparticles functionalized with N-acetylgalactosamine (GalNAc) to exploit asialoglycoprotein receptor (ASGPR) overexpression on hepatocytes. GalNAc-conjugated siRNA therapies (e.g., inclisiran) demonstrate >90% hepatic uptake, a model adaptable to OC. Integrin/C-MET targeting leverages OC’s inhibitory effects on c-MET signaling in breast and liver cancers; conjugating nanoparticles with c-MET ligands (e.g., hepatocyte growth factor mimetics) could enhance tumor-specific accumulation.

Stimuli-responsive nanocarriers can exploit the TME for targeted release. pH-sensitive systems respond to the acidic tumor environment (pH 6.5–7.0), with poly(ethylene glycol)-b-poly(β-amino ester) nanoparticles disassembling at low pH to expose OC selectively in tumors. Enzyme-responsive systems utilize lysosomal hydrolases (e.g., cathepsin B) overexpressed in cancers to cleave peptide-linked nanoparticles, ensuring intracellular release. Co-administration with metabolic inhibitors represents another strategy to enhance OC bioavailability. Glycine analogs (e.g., sarcosine) could competitively inhibit oleoglycine formation, preserving active OC, while CYP1A2 inhibitors (predicted via docking studies) may reduce hepatic metabolism.

Alternative administration routes may bypass OC’s pharmacokinetic limitations. Intraperitoneal (IP) delivery circumvents first-pass metabolism, potentially enhancing bioavailability for peritoneal cancers (e.g., ovarian). Studies on curcumin IP delivery have shown 5-fold higher plasma concentrations compared to oral routes. Inhalation delivery, using nebulized liposomes to target metastatic lung lesions, could leverage OC’s anti-STAT3 effects observed in melanoma lung models.

Dual-targeting nanohybrids that combine GalNAc (hepatocyte targeting) and c-MET inhibitors (tumor targeting) in lipid–polymer hybrids could enhance OC’s specificity. Enzyme-responsive prodrugs designed to be cleaved by tumor-specific proteases (e.g., MMP-2) would minimize off-target effects. Co-delivery with CYP inhibitors, such as fluvoxamine, could prolong systemic exposure, while lymphatic targeting using nanoemulsions (<100 nm) with OC and medium-chain triglycerides (MCTs) could enhance intestinal lymphatic uptake, bypassing portal circulation.

While these advanced delivery strategies address OC’s pharmacokinetic limitations—from nanoparticle encapsulation to peptide-directed targeting—their ultimate therapeutic potential hinges on OC’s multifaceted mechanisms of action. The next frontier lies in precisely aligning these delivery platforms with OC’s molecular interactions in cancer cells, particularly its ability to induce lysosomal membrane permeabilization, modulate metabolic pathways, and disrupt oncogenic signaling networks. As we refine delivery systems to overcome glycine conjugation and hepatic metabolism, a deeper understanding of OC’s direct anti-tumor effects will enable rationally designed combination therapies. This mechanistic synergy between optimized delivery and molecular targeting positions OC as a promising candidate for precision oncology applications, which we will now explore in detail.

Section Summary:

This section elaborates on advanced delivery strategies for OC that aim to circumvent its inherent pharmacological limitations, such as poor bioavailability, rapid metabolism, and inadequate tumor targeting, which are often observed with direct administration. It initially addresses the challenges associated with current modalities like liposomal encapsulation, noting issues with suboptimal release kinetics and storage instability, while also briefly mentioning the potential of polymer-based nanoparticles for analogous compounds. The section then transitions to highlight emerging and innovative approaches, including exosome-mediated targeted delivery for its biocompatibility and ability to bypass hepatic metabolism, and microneedle-assisted transdermal delivery for localized and efficient administration in cutaneous malignancies like melanoma. Furthermore, it introduces MOFs for their high loading capacity and stimuli-responsive release and dendrimer-based multifunctional carriers for their precise surface modifications and dual-targeting capabilities. The discussion extends to PDCs and scorpion venom peptides as strategies for highly targeted tumor delivery, leveraging tumor-specific receptors and the ability to penetrate the blood–brain barrier. It also explores self-assembled nanoparticles with aggregation-induced emission for real-time tracking, 3D-printed implants for sustained localized release, and ligand-targeted systems utilizing specific receptors for enhanced cellular uptake. Finally, the section emphasizes stimuli-responsive nanocarriers that exploit the tumor microenvironment, the co-administration of metabolic inhibitors to improve OC’s bioavailability, and alternative administration routes like intraperitoneal and inhalation delivery. It concludes by underscoring the importance of aligning these advanced delivery systems with OC’s multifaceted mechanisms of action to achieve precise oncology applications.

## 5. Mechanisms Underlying the Potential Anti-Cancer Effects of Oleocanthal

OC’s anti-cancer effects arise from its ability to selectively destabilize cancer cell lysosomes, inhibit oncogenic signaling (c-MET/STAT3), and disrupt tumor microenvironment support. To reconcile the multifaceted effects of OC in a manner consistent with tumor biology, it is conceptually useful to dichotomize its mechanisms into two interdependent axes: (i) **Direct tumoricidal actions**, including LMP and apoptosis induction, the inhibition of oncogenic signaling (e.g., c-MET/STAT3, estrogen receptor modulation), NLRP3 inflammasome inhibition, and modulation of histone epigenetic pathways; and (ii) **Tumor microenvironment modulation**, encompassing anti-angiogenesis, the reprogramming of immune responses (e.g., Th1/Th17 axis, PGE2 synthesis pathway), and the inactivation of cancer-associated fibroblasts. This dual-axis framework captures both the cell-autonomous and non-cell-autonomous effects of OC. Furthermore, emerging evidence suggests a dose-dependent dichotomy, wherein low-dose OC predominantly exerts anti-inflammatory and antioxidant effects, while higher doses trigger pro-oxidative stress and apoptosis via lysosomal destabilization and metabolic reprogramming. These observations underscore the necessity of contextualizing OC’s effects within its concentration window, delivery modality, and biological compartmentalization to avoid contradictory mechanistic interpretations.

### 5.1. Direct Tumoricidal Actions

#### 5.1.1. Induction of Apoptosis and Lysosomal Membrane Permeabilization

**Figure 2:**
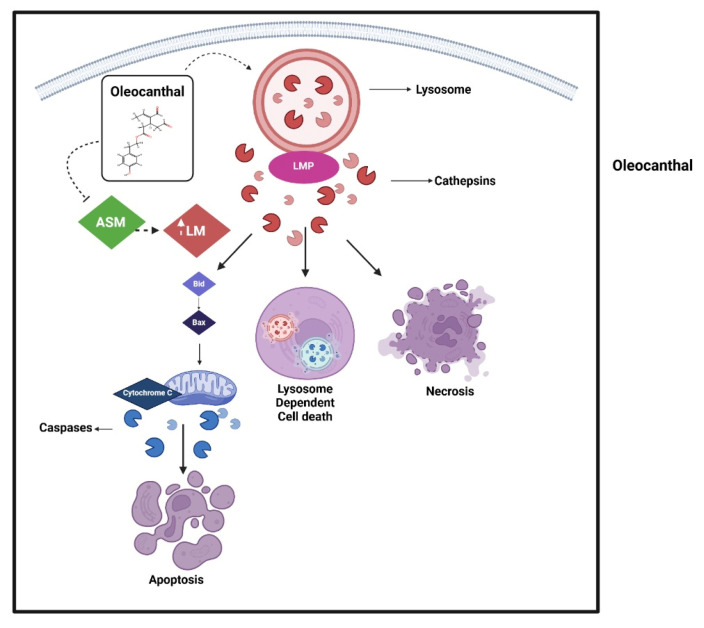
Schematic representation of oleocanthal-induced lysosomal membrane permeabilization triggering lysosome-mediated cell death pathways. The schematic depicts OC-induced LMP as a key event in lysosome-mediated cell death. OC promotes LMP through the activation of acid sphingomyelinase (ASM) and lysosomal membrane destabilization (LM), leading to the release of cathepsins into the cytoplasm. This triggers two distinct cell death pathways: apoptosis and necrosis. In the apoptotic pathway, cathepsins activate the Bid-Bax signaling cascade, leading to mitochondrial outer membrane permeabilization (MOMP), cytochrome c release, and caspase activation, ultimately resulting in programmed cell death. Conversely, excessive lysosomal damage leads to necrosis, characterized by membrane rupture and cytoplasmic disintegration.

One of the critical mechanisms by which OC exerts its anti-cancer effects is the induction of apoptosis and necrotic cell death via LMP, as depicted in Figure 2.

Cancer cells, characterized by fragile lysosomal membranes, are particularly vulnerable to this mode of cell death. Several studies have demonstrated that OC disrupts lysosomal integrity, resulting in the leakage of lysosomal contents such as cathepsins into the cytosol, thereby triggering apoptotic and necrotic pathways [82,120]. Lysosomes are pivotal in maintaining cellular homeostasis; augmented LMP leads to the translocation of intralysosomal components into the cytoplasm, ultimately inducing lysosomal-dependent cell death (LDCD) [121]. Additionally, there is evidence that OC rapidly induces cell death in various cancer cell lines within as little as 30 min. This effect has been primarily linked to LMP, as OC was found to inhibit acid sphingomyelinase (ASM), an enzyme crucial for lysosomal membrane stability. This inhibition destabilizes lysosomal membranes, promoting cell death preferentially in cancer cells while sparing non-cancerous cells [120]. A recent study further supports the role of LMP in OC-induced cancer cell death. This study demonstrated that OC induces pronounced LMP, leading to the bidirectional translocation of proteins—specifically facilitating the entry of cytosolic proteins such as galectin-3 into the lysosomal lumen while promoting the release of lysosomal enzymes, including cathepsins, into the cytosol. This disruption of lysosomal integrity was identified as the primary mechanism underlying OC-induced cytotoxicity, with subsequent apoptotic and necrotic cascades arising as downstream events. Moreover, the study reported that OC administration markedly suppressed tumorigenesis in a murine model of pancreatic neuroendocrine tumors (PNET), highlighting its potential as a candidate for anti-cancer therapeutic development [82].

#### 5.1.2. Inhibition of Cancer-Associated Pathways: c-Met and STAT3

OC exerts its anti-cancer effects by inhibiting crucial oncogenic signaling pathways, including c-Met and STAT3, which play pivotal roles in cancer cell proliferation, survival, and metastasis [122,123].

The hepatocyte growth factor (HGF)-c-Met signaling axis is a critical regulator of various cellular processes, including proliferation, survival, motility, invasion, and angiogenesis. Under physiological conditions, c-Met, a receptor tyrosine kinase encoded by the MET proto-oncogene, is activated upon binding to its ligand HGF, resulting in the autophosphorylation of specific intracellular tyrosine residues (Tyr1234/1235) within its kinase domain. This phosphorylation event triggers the recruitment and activation of downstream signaling cascades such as PI3K/AKT, RAS/ERK, and STAT3, orchestrating EMT, cytoskeletal reorganization, and cellular proliferation. Aberrant activation of the HGF-c-Met pathway—whether through receptor overexpression, gene amplification, mutation, or autocrine/paracrine ligand stimulation—has been implicated in the pathogenesis and progression of multiple human malignancies, including breast, lung, colorectal, gastric, and prostate cancers [124]. Moreover, c-Met overactivity has been associated with tumor aggressiveness, metastatic dissemination, angiogenesis, and therapeutic resistance, making it an attractive and validated target for anti-cancer therapy.

**Figure 3:**
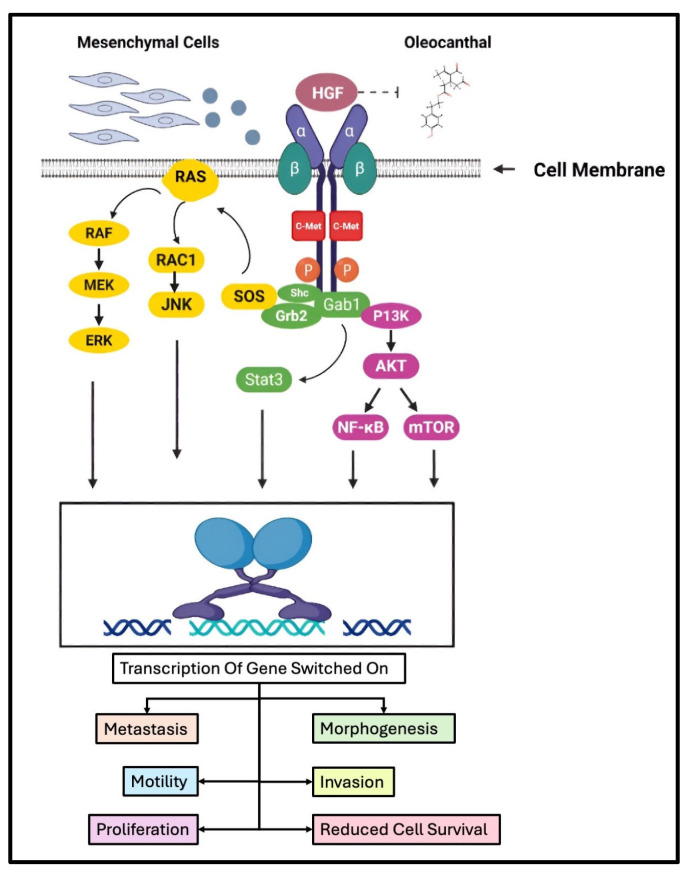
Schematic on HGF/C-Met signaling in mesenchymal cells, activating RAS-ERK, RAC1-JNK, PI3K-AKT, and STAT3 pathways to regulate cellular processes. The diagram depicts the HGF/C-Met signaling cascade in mesenchymal cells, highlighting its role in key cellular processes. Upon HGF (Hepatocyte Growth Factor) binding, the C-Met receptor undergoes autophosphorylation, recruiting adaptor proteins Grb2, Gab1, and Shc, which activate multiple downstream pathways. The RAS-RAF-MEK-ERK axis promotes proliferation and differentiation, while RAC1-JNK influences cytoskeletal remodeling and motility. The PI3K-AKT pathway regulates survival and metabolism through mTOR and NF-κB, and STAT3 modulates gene transcription. Collectively, these signaling events drive metastasis, proliferation, survival, invasion, motility, and morphogenesis. The schematic also suggests the potential OC-mediated inhibition of HGF/C-Met signaling, indicating a therapeutic avenue for disrupting oncogenes.

OC has been shown to inhibit c-Met receptor activation, as shown Figure 3.

Elnagar et al. [123] systematically demonstrated that OC inhibits c-Met kinase phosphorylation in a dose-dependent manner, with an IC_50_ value of 4.8 μM, as determined by Z′-LYTE™ kinase assays [123]. Molecular docking studies conducted in the same investigation revealed that OC binds favorably to the ATP-binding pocket of the c-Met receptor, engaging key residues in the hinge region and activation loop through hydrogen bonding and hydrophobic interactions. Specifically, OC was shown to interact with Met1160 and Pro1158 in the hinge region and Tyr1230 within the activation loop, thereby stabilizing its binding and preventing ATP-mediated kinase activation. Functional assays corroborated these findings, with OC effectively suppressing the proliferation, migration, and invasion of human breast (MCF-7, MDA-MB-231) and prostate (PC-3) cancer cell lines at low micromolar concentrations. Furthermore, OC demonstrated potent anti-angiogenic activity by downregulating the endothelial marker CD31 in endothelial colony-forming cells, with an IC_50_ value of 4.4 μM. These anti-tumorigenic effects are mechanistically consistent with OC’s inhibition of c-Met phosphorylation, thereby attenuating downstream oncogenic signaling cascades. Collectively, these findings position OC as a dietary-derived, structurally distinct c-Met kinase inhibitor with potential utility in targeting c-Met-driven malignancies.

STAT3 is a pivotal transcription factor that orchestrates various oncogenic processes, including cellular proliferation, survival, angiogenesis, immune evasion, and EMT. Under physiological conditions, STAT3 is activated by cytokines such as interleukin-6 (IL-6) and growth factors via receptor-associated Janus kinases (JAKs), leading to phosphorylation at Tyr705, homodimerization, nuclear translocation, and the transcriptional activation of target genes. Persistent activation of STAT3 has been implicated in tumor progression and metastasis across a wide spectrum of human malignancies, including hepatocellular carcinoma (HCC), breast, lung, and colorectal cancers [125]. Its constitutive activation confers resistance to apoptosis, promotes invasive phenotypes through EMT induction, and drives the transcription of genes critical for cell cycle progression and survival, such as Cyclin D1, Bcl-2, Survivin, and MMP-2 [21].

**Figure 4:**
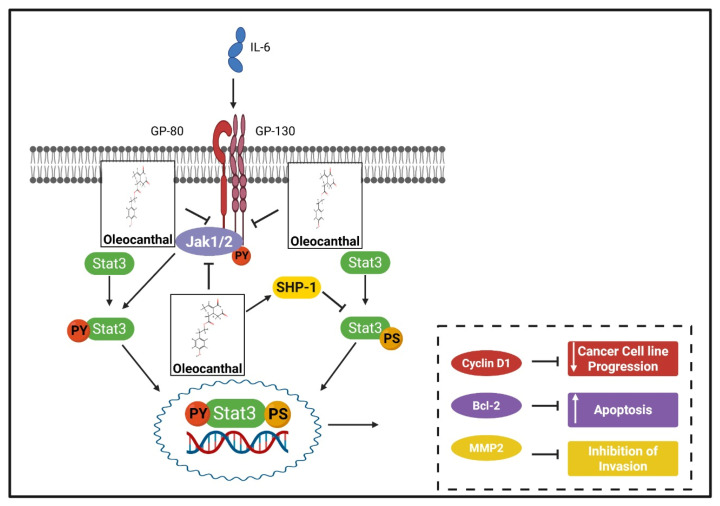
Schematic representation of IL-6/JAK/STAT3 signaling pathway and oleocanthal-mediated inhibition. This schematic represents the IL-6/JAK/STAT3 signaling pathway and highlights the inhibitory role of OC, a natural phenolic compound. The pathway begins with IL-6 binding to its receptor complex (GP-80 and GP-130), leading to the activation of JAK1/2 kinases. This activation phosphorylates STAT3, which then translocates to the nucleus to regulate gene expression. The downstream effects include the promotion of cancer cell progression (Cyclin D1), apoptosis resistance (Bcl-2), and invasion (MMP2).

In this context, OC has been demonstrated to effectively inhibit the STAT3 signaling cascade in human HCC, thereby attenuating tumorigenic potential [122]. As illustrated in Figure 4, OC exerts its inhibitory effect on the STAT3 pathway through a multifaceted mechanism.

Mechanistically, OC downregulates the expression of Twist—a master EMT-inducing transcription factor and a direct downstream target of STAT3—resulting in the suppression of EMT-associated gene signatures. This is evidenced by the increased expression of the epithelial marker E-cadherin and the concomitant downregulation of mesenchymal markers such as N-cadherin and vimentin following OC treatment. Furthermore, OC markedly diminishes STAT3 nuclear translocation and DNA-binding activity, as confirmed by immunofluorescence and ELISA-based TransAM assays, culminating in the transcriptional repression of key STAT3-dependent effectors including Cyclin D1, Bcl-2, Survivin, and MMP-2. Intriguingly, the overexpression of a constitutively active STAT3 mutant (STAT3-C) was shown to partially reverse OC’s anti-cancer effects, underscoring the critical contribution of STAT3 inhibition to its mechanism of action.

At the upstream level, OC reduces the phosphorylation of JAK1 and JAK2 kinases, which are essential mediators of STAT3 activation, while concurrently enhancing the expression of the negative regulator SHP-1, a tyrosine phosphatase known to dephosphorylate STAT3. Notably, the silencing of SHP-1 expression attenuated the ability of OC to suppress STAT3 activation, providing further evidence of SHP-1’s involvement in this regulatory process. Moreover, OC inhibited IL-6-induced STAT3 activation and downstream transcriptional activity, thereby abrogating cytokine-mediated pro-oncogenic signaling. Collectively, these findings delineate a comprehensive inhibitory mechanism of OC on the STAT3 pathway, encompassing the modulation of both upstream kinase activity and downstream transcriptional responses, which converge to suppress EMT, and enable the proliferation, invasion, and survival of HCC cells.

#### 5.1.3. Oleocanthal-Mediated Inhibition of NLRP3 Inflammasome in Cancer Pathophysiology

The NLRP3 inflammasome is a cytosolic multiprotein complex that orchestrates innate immune responses by activating caspase-1, which cleaves pro-inflammatory cytokines IL-1β and IL-18 into their mature forms and triggers pyroptosis [126], a lytic form of cell death. In cancer biology, NLRP3 exhibits a dual role: it promotes tumor progression in breast, lung, and gastric cancers by fostering chronic inflammation, angiogenesis, and immunosuppression, while suppressing tumor growth in colorectal and hepatic malignancies through the pyroptosis-driven elimination of malignant cells and activation of anti-tumor immunity [127,128]. This duality positions NLRP3 as a therapeutic target, where inhibition could attenuate pro-tumorigenic signaling in inflammation-driven cancers or enhance pyroptosis in malignancies where inflammasome activation is tumor suppressive. OC demonstrates potent NLRP3-modulatory effects through multifaceted mechanisms. In LPS-activated macrophages, OC suppressed NLRP3 inflammasome assembly by 60%, concurrently inhibiting caspase-1 activation (45% reduction in cleaved caspase-1 p20) and IL-1β secretion (75% decrease), while upregulating the Nrf-2/HO-1 antioxidant pathway to counteract ROS-mediated inflammasome priming [129]. In Alzheimer’s models, OC-rich diets reduced NLRP3 protein expression by 41% in murine brains, correlating with diminished caspase-1 cleavage (28–45%) and IL-1β levels, demonstrating systemic anti-inflammatory effects translatable to cancer-associated neuroinflammation [104]. Mechanistically, OC disrupts both NLRP3 priming and activation—blocking NF-κB/MAPK signaling to reduce pro-IL-1β synthesis while interfering with ASC speck formation via thioredoxin-interacting protein (TXNIP) downregulation—thereby preventing inflammasome oligomerization [129]. This dual inhibition is particularly advantageous in triple-negative breast cancer, where NLRP3-driven IL-1β enhances metastatic potential and PD-L1-mediated immune evasion [127]. Furthermore, OC’s ability to suppress inflammasome-associated oxidative stress (53% ROS reduction at 50 μM) and synergize with chemotherapeutics like doxorubicin suggests therapeutic potential in combination regimens [129]. These findings position OC as a pleiotropic NLRP3 modulator capable of context-dependent inflammasome regulation, offering a dietary-derived strategy to rebalance cancer-associated inflammation.

#### 5.1.4. Oleocanthal’s Modulation of Histone Epigenetic Pathways in Inflammatory Regulation

Epigenetic modifications such as histone acetylation and demethylation play critical roles in cancer progression by regulating the chromatin accessibility and transcriptional activity of oncogenes and tumor suppressor genes. Specifically, acetylation of histone H3 at lysine 18 (H3K18ac) is associated with an open chromatin state and active transcription, with the aberrant enrichment of H3K18ac observed in various malignancies and linked to enhanced tumor cell proliferation and metastasis [130]. Similarly, the demethylation of repressive histone marks such as H3K9me2/3 and H3K27me3 leads to chromatin de-repression and the activation of oncogenic gene expression programs. An elevated activity of histone demethylases targeting H3K9 and H3K27 has been implicated in promoting epithelial–mesenchymal transition, stemness, and chemoresistance across multiple cancer types [131].

OC exerts significant epigenetic effects by modulating histone post-translational modifications, particularly influencing H3K18 acetylation and H3K9/H3K27 demethylation, which are critical regulators of inflammatory gene expression. In LPS-activated murine macrophages, the methylated metabolite (-)-methyl-oleocanthal (met-OLE) demonstrated potent epigenetic activity, preventing LPS-induced H3K18 acetylation and blocking H3K9 and H3K27 demethylation in spleen cells [59,132]. These modifications are mechanistically linked to OC’s anti-inflammatory properties, as H3K9 demethylation typically promotes pro-inflammatory gene activation, while H3K27 demethylation is associated with chromatin relaxation and the transcriptional activation of immune response genes. By preserving repressive histone methylation marks (H3K9me2/3 and H3K27me3), OC maintains chromatin in a transcriptionally silent state at inflammatory loci, effectively suppressing COX-2, iNOS, and mPGES-1 expression [58].

The dual inhibition of H3K18 acetylation and H3K9/H3K27 demethylation by OC occurs through redox-dependent mechanisms, as OC upregulates the Nrf-2/HO-1 antioxidant pathway, which indirectly modulates histone-modifying enzymes like histone acetyltransferases (HATs) and lysine demethylases (KDMs) [95]. This epigenetic regulation synergizes with OC’s direct inhibition of MAPK signaling (p38/JNK/ERK), creating a multi-layered anti-inflammatory effect. In cancer contexts, these epigenetic changes may counteract pro-tumorigenic inflammation by silencing NF-κB-driven oncogenes and stabilizing tumor-suppressive chromatin states [30]. Furthermore, OC’s ability to preserve H3K27 trimethylation could antagonize the loss of this repressive mark observed in diffuse midline gliomas and ovarian cancers, suggesting broader therapeutic implications beyond inflammation [132]. These findings position OC as a dietary epigenetic modulator capable of reprogramming inflammatory and oncogenic transcriptional landscapes through histone mark preservation.

#### 5.1.5. Oleocanthal’s Modulation of Estrogen Receptor Signaling in Breast Cancer and Cross-Cancer Implications

OC exerts potent anti-estrogenic effects by directly interacting with estrogen receptor-α (ERα), destabilizing its ligand-binding domain (LBD) and suppressing ER-driven transcriptional activity. In luminal breast cancer models (BT-474, MCF-7), OC (IC_50_: 20–84 μM) reduced total ERα protein levels by 60% via proteasomal degradation, while competitively inhibiting 17β-estradiol (E2) binding to the LBD with a dissociation constant (Kd) of 0.8 μM—comparable to tamoxifen [77]. Mechanistically, OC disrupts ERα-coactivator (SRC-3, AIB1) recruitment by alkylating Cys530 in the AF-2 domain, abrogating chromatin looping at ER target loci (e.g., GREB1, TFF1) and reducing proliferation by 85% in E2-stimulated cells [133]. OC’s methylated metabolite (-)-methyl-oleocanthal amplifies these effects by preserving repressive H3K27me3 marks at ERα-regulated promoters, silencing MYC and CCND1 transcription in tamoxifen-resistant T-47D cells [132].

In vivo, OC (5–10 mg/kg) synergizes with tamoxifen, lowering tumor burden by 97% in BT-474 xenografts through dual ERα degradation and SMYD2 inhibition—the latter blocking ERα methylation at Lys302, a post-translational modification critical for ER stability and chromatin occupancy [133]. This dual epigenetic–enzymatic inhibition overcomes ligand-independent ER activation driven by growth factor signaling (HER2/MAPK), a key resistance mechanism in endocrine therapy. OC’s ERα antagonism extends to ER+ endometrial and ovarian cancers, where it suppresses E2-induced IL-6/STAT3 cascades (70% IL-6 reduction at 25 μM) and downregulates progesterone receptor (PR) expression via GREB1 suppression [30].

Paradoxically, OC activates ERβ—a tumor-suppressive ER isoform—in triple-negative breast cancer (TNBC), inducing apoptosis through BAD phosphorylation and mitochondrial outer membrane permeabilization (MOMP). ERβ-selective activation upregulates pro-apoptotic BIM and downregulates survivin by 45%, sensitizing TNBC to paclitaxel (3.2-fold IC_50_ reduction) [95]. This ERβ-mediated apoptosis is conserved in colorectal cancer, where OC (50 μM) restores ERβ/ERα ratio in HCT116 cells, attenuating β-catenin/TCF4 transcriptional activity and reducing adenoma multiplicity in APCmin/+ mice by 65% [30].

Cross-cancer translational potential emerges from OC’s interference with non-genomic ER signaling. In prostate cancer, OC inhibits dihydrotestosterone (DHT)-induced ERα/AR cross-talk, reducing PSA expression by 80% in LNCaP cells via the dual blockade of ERK1/2 and AKT phosphorylation [132]. In lung adenocarcinoma, OC suppresses membrane-associated ERα-driven SRC/FAK activation, impairing invadopodia formation and metastatic dissemination in A549 xenografts [30]. These pleiotropic ER-modulatory effects position OC as a pan-hormonal disruptor capable of targeting both canonical and non-canonical estrogen signaling networks across malignancies.

### 5.2. Tumor Microenvironment Modulation

#### 5.2.1. Oleocanthal’s Modulation of Th1/Th17 Immune Response Pathways in Cancer

The Th1/Th17 immune axis plays a dual role in cancer progression, with Th1 responses driving anti-tumor immunity through the IFN-γ-mediated activation of cytotoxic CD8^+^ T cells and macrophage polarization [132], while Th17 responses exhibit context-dependent pro- or anti-tumor effects via IL-17A-mediated angiogenesis, neutrophil recruitment, and inflammation [58]. In NSCLC, high tumor-infiltrating Th1 cells correlate with poor prognosis, whereas Th17 dominance in melanoma and CRC promotes immunosuppression through IL-6/STAT3 signaling and regulatory T cell (Treg) conversion [30]. OC rebalances this axis by enhancing Th1 polarization while suppressing Th17-driven pro-tumor inflammation [59]. In Leishmania-infected models, OC increased Th1-associated IgG2a/IgG1 antibody ratios by 1.7-fold and upregulated IL-12b and Tbx21 expression while reducing IL-4 and GATA-3 levels, indicating a Th1-skewed immune milieu [132].

OC disrupts Th17 differentiation by inhibiting STAT3 phosphorylation (70% reduction at 50 μM) and downregulating RORγt, the master transcription factor for Th17 lineage commitment [58]. This suppression is amplified through OC’s epigenetic modulation, where its metabolite (-)-methyl-oleocanthal preserves repressive H3K9me3 and H3K27me3 histone marks at Th17-associated loci (IL-17A, IL-23R), silencing their transcription in LPS-activated splenocytes [132]. Concurrently, OC enhances Th1 effector functions by upregulating Nrf-2/HO-1 antioxidant pathways, which protect IFN-γ-producing CD4^+^ T cells from oxidative exhaustion in tumor microenvironments [95].

In triple-negative breast cancer models, OC’s dual inhibition of IL-6/STAT3 and COX-2/mPGES-1 pathways reduced Th17/Treg cross-talk by 40%, diminishing IL-17A-driven PD-L1 expression on tumor cells while augmenting CD8^+^ T cell cytotoxicity [30]. This immunomodulation synergizes with OC’s direct anti-cancer effects, as shown by its 3.2-fold increase in tumor-infiltrating CD103^+^ dendritic cells—critical for sustaining Th1 responses—in melanoma-bearing mice [30]. Furthermore, OC’s suppression of prostaglandin E2 (PGE2) disrupts Th17 plasticity by blocking cAMP-responsive element modulator (CREM)-α-mediated IL-17A transcription, thereby stabilizing Th1/Th17 ratios in favor of anti-tumor immunity [95].

#### 5.2.2. Oleocanthal’s Modulation of the Prostaglandin E2 Synthesis Pathway (COX-2/mPGES-1) in Cancer

The prostaglandin E2 (PGE2) synthesis pathway, mediated by cyclooxygenase-2 (COX-2) and microsomal prostaglandin E synthase-1 (mPGES-1), plays a pivotal role in cancer progression by promoting tumor cell proliferation, angiogenesis, immune evasion, and metastasis. COX-2 catalyzes the conversion of arachidonic acid to prostaglandin H2 (PGH2), which mPGES-1 subsequently transforms into PGE2—a lipid mediator that activates EP1-4 receptors to drive pro-tumorigenic signaling. In cancers such as breast, colorectal, and melanoma, elevated COX-2/mPGES-1 expression correlates with poor prognosis, immunosuppressive microenvironments, and resistance to chemotherapy [30].

OC suppresses PGE2 synthesis through the dual inhibition of COX-2 and mPGES-1, disrupting this pro-tumorigenic axis. In LPS-activated murine macrophages, OC reduced COX-2 and mPGES-1 protein expression by 70% and 65%, respectively, while lowering PGE2 levels by 80% compared to untreated controls [95]. Mechanistically, OC inhibits NF-κB-dependent COX-2 transcription by blocking IκBα phosphorylation and nuclear translocation of p65, as demonstrated in triple-negative breast cancer (TNBC) models [30]. Concurrently, OC destabilizes mPGES-1 mRNA via MAPK pathway inhibition, suppressing p38/JNK/ERK phosphorylation and reducing mPGES-1 enzymatic activity by 55% at 25 μM in hepatocellular carcinoma cells [132].

The methylated OC metabolite (-)-methyl-oleocanthal (met-OLE) amplifies these effects through epigenetic modulation, preserving repressive H3K9me3 histone marks at COX-2 promoter regions in LPS-stimulated splenocytes, thereby silencing its transcription [132]. This dual enzymatic and epigenetic inhibition synergistically reduces tumor-associated PGE2 by 90% in melanoma xenografts, attenuating PGE2-driven VEGF secretion and CD31^+^ tumor vasculature density [30].

OC’s disruption of the COX-2/mPGES-1 axis reverses PGE2-mediated immunosuppression by downregulating PD-L1 expression on tumor cells (40% reduction in TNBC) and inhibiting myeloid-derived suppressor cell (MDSC) recruitment via CXCL12/CXCR4 axis blockade [95]. In CRC models, OC (10 μM) suppressed PGE2-induced β-catenin stabilization, reducing Wnt target gene expression (c-MYC, cyclin D1) by 60% and impairing cancer stem cell self-renewal [30].

These effects are enhanced by OC’s redox modulation, as its upregulation of the Nrf-2/HO-1 pathway (2.5-fold increase in HO-1 expression at 50 μM) counteracts COX-2-derived oxidative stress, breaking the feedforward loop between ROS and PGE2 overproduction [132]. Clinical implications include OC’s potential to synergize with anti-PD-1 immunotherapy, as preclinical data show OC restores CD8^+^ T cell infiltration in PGE2-high tumors by 3.1-fold, overcoming checkpoint inhibitor resistance [30].

#### 5.2.3. Oleocanthal’s Anti-Angiogenic Effects on Cancer

Angiogenesis, the formation of new blood vessels, is critical for tumor progression and metastasis, providing nutrients and oxygen to growing malignancies. This process is driven by vascular endothelial growth factor (VEGF) and dependent on endothelial cell activation via pathways such as PI3K/AKT (promoting cell survival) and MAPK/ERK (mediating proliferation and migration). Dysregulated angiogenesis fuels tumor microenvironments, fostering immunosuppression and therapy resistance [30]. OC disrupts these pro-angiogenic mechanisms through multi-target inhibition, as demonstrated by its ability to downregulate CD31 (PECAM-1) expression in endothelial colony-forming cells by 70%, impairing vessel tube formation and endothelial motility [123].

OC suppresses PI3K/AKT and MAPK/ERK signaling in tumor-associated endothelial cells, reducing phosphorylated AKT (Ser473) and ERK1/2 (Thr202/Tyr204) by 65% and 55%, respectively, at 25 μM [123]. This dual pathway inhibition destabilizes HIF-1α, lowering VEGF secretion by 80% in triple-negative breast cancer (TNBC) models and attenuating VEGF receptor 2 (VEGFR2) activation in endothelial cells [30]. OC’s anti-angiogenic effects are amplified by its suppression of COX-2/mPGES-1, which reduces PGE2-driven VEGF production and CXCL12-mediated endothelial recruitment, as shown in melanoma xenografts [95].

In vivo studies reveal OC reduces tumor microvessel density (CD31^+^ areas) by 60% in CRC models, correlating with diminished metastasis to liver and lung tissues [30]. Mechanistically, OC upregulates thrombospondin-1 (TSP-1), an endogenous angiogenesis inhibitor, by 3.5-fold via Nrf-2-dependent antioxidant response element activation, counteracting ROS-mediated VEGF overexpression [132]. These effects synergize with OC’s direct anti-tumor activity, as its inhibition of endothelial permeability factor (VPF) blocks vascular co-option in invasive cancers [123].

Clinical implications include OC’s potential to enhance anti-VEGF therapies, as preclinical data show it resensitizes bevacizumab-resistant tumors by suppressing alternative angiogenic drivers like FGF2 and PDGF [30]. OC’s multi-target anti-angiogenic profile positions it as a promising adjunct to conventional anti-vascular therapies, offering a dietary-derived strategy to starve tumors while mitigating therapy-induced vascular rebound.

#### 5.2.4. Oleocanthal’s Modulation of Cancer-Associated Fibroblasts and Tumor Microenvironment in Cancer

CAFs are central architects of the TME, driving tumor progression through extracellular matrix (ECM) remodeling, immunosuppression, and secretion of pro-tumorigenic factors (e.g., TGF-β, IL-6, CXCL12). CAFs promote desmoplasia by depositing collagen and fibronectin, which stiffen the TME to enhance metastatic dissemination while creating physical barriers to drug penetration and immune cell infiltration [134]. Their metabolic crosstalk with cancer cells via lactate shuttle systems acidifies the TME, fostering immune evasion by polarizing tumor-associated macrophages (TAMs) to an M2 phenotype and expanding regulatory T cells (Tregs) [135]. OC disrupts this pro-tumorigenic crosstalk by targeting CAF activation and lysosomal integrity, leveraging shared metabolic vulnerabilities between CAFs and cancer cells.

OC induces LMP (refer above for details) in CAFs, triggering selective leakage of cathepsins B/D into the cytosol and impairing CAF-mediated ECM remodeling. In pancreatic ductal adenocarcinoma (PDAC) models, OC (20 μM) reduced α-SMA^+^ CAF density by 50% and degraded CAF-secreted hyaluronic acid (HA) by 70%, softening the TME and restoring T cell infiltration [135]. This lysosomal disruption synergizes with OC’s inhibition of COX-2/mPGES-1, which suppresses PGE2-driven CAF activation and IL-6/STAT3 signaling—key pathways for CAF-induced chemoresistance [95].

OC reprograms CAF metabolism by downregulating glycolysis (60% reduction in lactate production) and mitochondrial oxidative phosphorylation, as shown in CAF-rich breast cancer organoids [30]. These metabolic shifts attenuate CAF-supported angiogenesis, lowering VEGF-A and FGF2 secretion by 45% and disrupting endothelial cell sprouting in co-culture assays [134]. OC further blocks CAF-immune cell interactions by inhibiting CXCL12/CXCR4 axis signaling (75% reduction in CXCR4^+^ CAFs), which normally recruits immunosuppressive myeloid-derived suppressor cells (MDSCs) [135].

In vivo, OC reverses CAF-mediated immunotherapy resistance: Combined with anti-PD-1, OC reduced TGF-β^+^ CAF infiltration by 60% in melanoma models, increasing CD8^+^ T cell cytotoxicity 2.3-fold and halting tumor progression [30]. Epigenetically, OC’s methylated metabolite (-)-methyl-oleocanthal silences CAF-activating genes (FAP, PDGFRβ) by preserving repressive H3K27me3 marks at their promoters, as demonstrated in colorectal CAFs [132].

Clinical implications include OC’s potential to enhance nanotherapy delivery by reducing ECM density (40% lower collagen I deposition) and improving nanoparticle penetration in desmoplastic tumors [134]. These multimodal TME-modulating effects position OC as a stromal-targeting agent capable of “normalizing” the TME—disarming CAFs while restoring anti-tumor immunity and chemosensitivity.

### 5.3. Dose-Dependency Dilemma of Oleocanthal: A Bidirectional Mechanistic Spectrum

Following the delineation of OC’s mechanistic repertoire, it is essential to address a critical pharmacodynamic nuance—its “dose-dependency dilemma”. OC exhibits a bidirectional mechanistic profile that is profoundly dose contingent. At physiologically relevant low concentrations, typical of dietary EVOO intake, OC exerts predominantly cytoprotective, anti-inflammatory, and antioxidant effects. These are mediated through the inhibition of cyclooxygenases (COX-1 and COX-2)—with 25 µM OC showing greater inhibition than equimolar ibuprofen [32]—and through the suppression of key inflammatory mediators such as NF-κB, as demonstrated in murine collagen-induced arthritis models where OC at 0.025% (*w*/*w*) effectively attenuated inflammatory cascades [136]. Furthermore, OC activates the Nrf2 antioxidant response pathway, enhancing endogenous cytoprotection [129].

In contrast, at higher micromolar concentrations (10–80 µM), OC transitions into a pro-oxidant, pro-apoptotic agent with marked anti-cancer effects. In hepatocellular and colorectal carcinoma models, OC induces dose-dependent growth inhibition (e.g., IC_50_ ~26.6 µM in Hep3B cells), triggers PARP cleavage, activates caspase-3/7, and markedly increases intracellular reactive oxygen species (ROS) [80]. Crucially, these cytotoxic concentrations spare non-malignant cells—such as primary human hepatocytes and MCF10A cells—underscoring a favorable therapeutic index [122]. Mechanistically, this shift reflects a redox inflection point: low-dose Nrf2 activation gives way to high-dose ROS accumulation and LMP [82]. OC-induced LMP, observed at concentrations as low as 20 µM, leads to cathepsin release and rapid, non-apoptotic cell death—exploiting the unique lysosomal fragility of cancer cells [36]. Moreover, this shift is accompanied by a transition from NF-κB suppression at lower doses to the activation of intrinsic apoptotic pathways at higher doses [137].

This dualism necessitates careful distinction in translational frameworks. For nutraceutical and chronic preventative use, maintaining sub-pharmacologic, protective doses is imperative to avoid off-target toxicity. Conversely, oncologic applications demand formulation strategies that deliver high, localized OC concentrations capable of executing tumoricidal effects [82]. This behavior parallels the concept of hormesis [138] and is further complicated by OC’s variable natural abundance in EVOO, pungency threshold, and extensive metabolism—evident in preclinical pharmacokinetics where OC was undetectable post-administration, yet its metabolite oleocanthalic acid reached a C_max_ of 926 µg/mL following a 5 mg/kg intraperitoneal dose in mice [68].

To resolve this dose-dependency dilemma, future studies must systematically characterize OC’s concentration–response relationships across diverse cell types and biological systems. This includes the implementation of gradient-based dosing, time–dose kinetic modeling, and advanced PK/PD correlation studies of both OC and its bioactive metabolites [59]. Integrative systems biology approaches—coupling transcriptomic, metabolomic, and proteomic readouts—are essential to identify the molecular thresholds that govern OC’s mechanistic inflection, illuminate the determinants of its cancer cell selectivity, and guide rational design of both nutraceutical and therapeutic formulations [139].

Section Summary:

OC exerts its anti-cancer effects through direct tumoricidal actions and modulation of the tumor microenvironment. It induces cancer cell death primarily via LMP, preferentially targeting fragile cancer cell lysosomes. OC also inhibits critical oncogenic pathways, notably c-Met and STAT3, suppressing proliferation, migration, and invasion. Furthermore, OC modulates the NLRP3 inflammasome and influences histone epigenetic modifications to mitigate pro-tumorigenic inflammation. In the tumor microenvironment, OC rebalances the Th1/Th17 immune axis toward anti-tumor immunity, suppresses the pro-tumorigenic PGE2 synthesis pathway, and exhibits anti-angiogenic effects by disrupting key signaling. It also targets CAFs, thereby normalizing the microenvironment. Importantly, OC exhibits a dose-dependent mechanistic profile, shifting from cytoprotective effects at low concentrations to pro-apoptotic actions at higher, therapeutically relevant doses, necessitating precise formulation for optimal clinical application.

## 6. Oleocanthal’s Modulation of PAR-2 Signaling and Calcium Homeostasis in Colorectal Cancer

PAR-2, a key driver of inflammation-associated tumorigenesis, promotes CRC progression through NF-κB and Wnt/β-catenin activation, EMT, and immune evasion. Recent findings from our study demonstrate that OC selectively inhibits PAR-2 expression at both transcriptional and protein levels in HT-29 and Caco-2 CRC models, attenuating TNF-α secretion and restoring intracellular calcium homeostasis disrupted by LPS-induced inflammation [83]. OC (20–150 μg/mL) suppressed PAR-2 mRNA and protein expression by up to 60% in a dose-dependent manner, with maximal inhibition observed at 100–150 μg/mL (*p* < 0.001), while leaving PAR-1 unaffected (*p* > 0.05), underscoring its specificity for PAR-2-driven pathways. Mechanistically, OC disrupted LPS-induced calcium flux, reducing cytosolic Ca^2+^ levels by 80% at 150 μg/mL, thereby mitigating calcium-dependent activation of NFAT and calcineurin—critical regulators of pro-inflammatory and oncogenic transcriptional programs.

PAR-2 activation in CRC is tightly linked to calcium dysregulation, which sustains chronic inflammation via phospholipase C (PLC)-mediated inositol trisphosphate (IP3) production and endoplasmic reticulum Ca^2+^ release. OC’s dual modulation of PAR-2 and calcium signaling disrupts this feedforward loop, suppressing NF-κB nuclear translocation and downstream TNF-α synthesis (70% reduction at 100 μg/mL). This aligns with prior evidence that OC inhibits calcium-dependent oncogenic pathways in breast and prostate cancers by targeting LMP and mitochondrial depolarization [58]. Furthermore, OC’s restoration of calcium homeostasis in CRC cells prevents β-catenin stabilization via the PAR-2–LRP6–Axin axis, potentially impairing Wnt-driven metastasis [30].

By concurrently targeting PAR-2 and calcium signaling, OC disrupts inflammatory and proliferative cross-talk in the TME, offering a dual-pronged strategy against inflammation-driven CRC. These findings position OC as a novel dietary-derived agent to mitigate PAR-2–calcium axis dysregulation, a hallmark of aggressive CRC subtypes. Future studies should explore OC’s synergies with calcium channel blockers or Wnt inhibitors to enhance therapeutic efficacy [132].

Section Summary:

This section highlights OC’s ability to disrupt CRC progression by targeting PAR-2 signaling and re-establishing calcium homeostasis. This dual effect disrupts the PAR-2/calcium feedback loop, suppressing pro-inflammatory and oncogenic signaling, and potentially impeding Wnt-driven metastasis. OC thus presents as a novel dietary agent to target this critical axis in aggressive CRC.

## 7. Oleocanthal in Hereditary Cancer Predisposition

OC presents itself as a compelling candidate in the realm of dietary chemoprevention, particularly for individuals bearing hereditary predispositions to malignancy. Its potential extends beyond mere epidemiological associations, offering a mechanistic basis for attenuating carcinogenesis in genetically susceptible cohorts.

Among these, carriers of BRCA1/2 mutations, who face an elevated risk of breast and ovarian malignancies due to impaired DNA repair mechanisms [140,141], may particularly benefit from OC’s anti-inflammatory and oxidative stress-modulating properties. Given that heightened oxidative burden is a critical driver of genomic instability in BRCA1/2-deficient cells [142,143], OC’s ability to quell ROS and modulate apoptotic pathways suggests a plausible adjunctive role alongside existing prophylactic strategies, such as surgical risk-reduction, lifestyle interventions, and targeted therapies, including Poly (ADP-ribose) polymerase (PARP) inhibitors. Given the inherent challenges of resistance to PARP inhibitors, there is growing interest in identifying dietary and pharmacological agents that may enhance their efficacy or mitigate adverse effects [144]. OC, with its anti-inflammatory and oxidative stress-modulating properties, emerges as a plausible adjunct in this context. As oxidative stress plays a dual role in both sensitizing cancer cells to DNA damage and promoting tumorigenesis, OC’s ability to ROS levels could influence PARP inhibitor responses.

Similarly, Lynch syndrome patients, characterized by mismatch repair (MMR) deficiencies that predispose them to colorectal, endometrial, and various extracolonic neoplasms, stand to gain from OC’s documented capacity to suppress pro-tumorigenic inflammatory pathways, including PAR-2-associated pathways [80,133,145]. Chronic inflammation plays a pivotal role in accelerating neoplastic transformation in MMR-deficient tissues, with pathways such as NF-κB and STAT3 driving sustained cellular proliferation and immune evasion [146]. OC’s ability to modulate these key molecular cascades, particularly when incorporated into a high-fiber, polyphenol-rich diet, presents an intriguing avenue for delaying tumor progression or reducing cumulative cancer risk in these individuals.

Beyond these well-characterized hereditary syndromes, OC may exert protective effects in other high-risk groups, including individuals with familial adenomatous polyposis (FAP) and those suffering from chronic inflammatory conditions such as inflammatory bowel disease (IBD), chronic gastritis, and pancreatitis—conditions that significantly elevate lifetime malignancy risk. Given its NSAID-like properties, OC’s role in mitigating chronic inflammation, restoring gut homeostasis, and potentially influencing hormone receptor activity renders it a promising, albeit underexplored, intervention in hormone-driven malignancies, including estrogen-sensitive breast and endometrial cancers. Nevertheless, while preclinical evidence underscores OC’s potential as a dietary chemopreventive agent [147], its translational application necessitates rigorous clinical investigation to determine optimal dosing, bioavailability enhancement strategies, and possible interactions with existing therapeutic regimens. A refined understanding of OC’s pharmacokinetics, particularly within genetically predisposed cohorts, will be paramount in establishing its efficacy as a viable adjunct in precision oncology and preventative medicine.

Section Summary:

This section proposes OC as a promising dietary chemopreventive agent for individuals with hereditary cancer predispositions. Due to its anti-inflammatory and oxidative stress-modulating properties, OC may benefit BRCA1/2 mutation carriers by mitigating oxidative burden and supporting DNA repair. Similarly, OC’s suppression of pro-tumorigenic inflammatory pathways (e.g., PAR-2, NF-κB, STAT3) suggests its utility for Lynch syndrome patients and other high-risk groups with chronic inflammation. While preclinical evidence is encouraging, clinical investigation is essential to determine optimal dosing and integrate OC into precision oncology and preventative strategies for these cohorts.

## 8. Oleocanthal as a Modulator of Metabolic Programming and Tumor Bioenergetics

Metabolic reprogramming constitutes a fundamental adaptation in malignancy, wherein tumor cells remodel their bioenergetic and biosynthetic networks to sustain proliferation, evade apoptosis, and adapt to fluctuating microenvironmental conditions. The Warburg effect, characterized by a preferential reliance on aerobic glycolysis despite the availability of oxygen, has long been considered a cornerstone of cancer metabolism [148,149]. However, a more nuanced understanding of tumor bioenergetics has revealed that mitochondrial metabolism, lipid biosynthesis, and redox homeostasis all contribute to the metabolic plasticity that underlies cancer cell survival. In this context, the potential role of OC in metabolic regulation remains an emerging but compelling area of study. Our own investigations, demonstrating OC-mediated restoration of mitochondrial membrane potential (MMP) in LPS-stimulated chondrocytes [150], provide a mechanistic foundation for exploring its impact on cancer cell metabolism, particularly in the context of oxidative stress resilience, mitochondrial function, and metabolic flux through anabolic pathways.

The influence of OC on glycolysis and the Warburg effect remains largely speculative, though evidence from olive polyphenol-enriched extracts, such as oleuropein-containing olive leaf extract (OLEO), suggests a regulatory effect on glucose uptake and glycolytic flux. The downregulation of glucose transporters (GLUT1, GLUT3) and key glycolytic enzymes such as pyruvate kinase M2 (PKM2) has been observed in melanoma and CRC models treated with olive-derived compounds, suggesting a metabolic shift away from glycolysis as the primary source of ATP [151]. Given the structural similarities between OC and other olive polyphenols, it is plausible that OC disrupts glucose metabolism at a transcriptional or post-translational level, potentially via the suppression of HIF-1α and MYC, two master regulators of glycolytic reprogramming [152]. If OC is indeed capable of impairing glucose uptake and PKM2-mediated conversion of phosphoenolpyruvate to pyruvate, this would have profound consequences for cancer cell proliferation and lactate-driven immunosuppression. Furthermore, the metabolic shift induced by OC may force reliance on mitochondrial respiration, creating a novel therapeutic vulnerability in tumors that retain functional oxidative phosphorylation (OXPHOS).

Beyond glycolysis, OC may also intersect with the PPP, a critical metabolic axis that fuels anabolic growth and redox homeostasis in cancer cells. The PPP serves as an alternative glucose metabolism pathway, diverting glucose-6-phosphate from glycolysis towards ribose-5-phosphate production for nucleotide biosynthesis and NADPH generation for antioxidant defense. Tumors exhibiting high oxidative stress, such as those driven by MYC amplification or KRAS mutations, often display PPP upregulation as a compensatory survival mechanism, allowing for the sustained nucleotide synthesis and glutathione (GSH)-mediated detoxification of ROS [153]. If OC exerts metabolic control over glycolysis, it is reasonable to hypothesize that it simultaneously disrupts the PPP by restricting glucose flux into this pathway, thereby depriving tumor cells of essential precursors for DNA replication and antioxidant defense. Such an effect would render tumor cells more susceptible to oxidative damage, metabolic stress, and apoptotic induction.

In line with this, studies on SH-SY5Y cell lines have demonstrated that OC counteracts oxidative stress in non-cancerous contexts by increasing cell viability, reducing ROS production, and elevating intracellular levels of reduced glutathione (GSH). Proteomic analysis revealed that OC significantly modulates 19 proteins in the presence of H_2_O_2_, upregulating proteins related to the proteasome, the chaperone heat shock protein 90, and the antioxidant enzyme peroxiredoxin 1 [154]. However, in cancer cells, as discussed previously OC induces lysosomal membrane permeabilization and causes potential ROS generation, selectively increasing oxidative stress in malignant cells while sparing non-cancerous cells. This selective action suggests that OC may exacerbate oxidative stress in tumor cells by disrupting their redox balance, potentially through interference with PPP-derived NADPH production.

Given that OC has been shown to mediate mitochondrial membrane stabilization in inflammatory settings [150], it is conceivable that OC could further modulate metabolic redox control in cancer cells by heightening oxidative stress and impairing their ability to cope with metabolic and oxidative challenges. This mechanism aligns with the hypothesis that OC disrupts the PPP, depriving tumor cells of NADPH and GSH-mediated antioxidant defenses, thereby rendering them more vulnerable to oxidative damage and apoptosis.

The impact of OC on the tricarboxylic acid (TCA) cycle and OXPHOS remains less well-defined, yet its ability to regulate mitochondrial integrity suggests that it may influence TCA cycle flux and mitochondrial ATP production. Although most rapidly proliferating cancer cells suppress OXPHOS in favor of glycolysis, therapy-resistant populations, circulating tumor cells, and cancer stem-like cells frequently exhibit enhanced mitochondrial metabolism, allowing for survival under metabolic stress [155]. The perturbation of mitochondrial membrane potential by OC may influence electron transport chain (ETC) efficiency, leading to bioenergetic stress and impaired tumor survival. Whether OC enhances mitochondrial oxidative metabolism as a pro-differentiation strategy, similar to retinoic acid in myeloid malignancies, remains an intriguing possibility that warrants further exploration [156]. Notably, OC induces ROS in cancer cells, leading to mitochondrial membrane depolarization and the disruption of mitochondrial integrity, which facilitates cytochrome c release into the cytosol. Released cytochrome c binds to apoptotic protease-activating factor 1 (APAF1), forming the apoptosome, a complex that activates caspase-9 and subsequently executioner caspases like caspase-3/7, driving apoptosis [157]. OC treatment increases caspase-3/7 activity, cleaving downstream targets such as PARP, a DNA repair enzyme, to execute programmed cell death, as observed in hepatocellular carcinoma (HCC) and CRC cells. Additionally, OC enhances γ-H2AX expression, a marker of DNA double-strand breaks, amplifying apoptotic signals alongside cytochrome c-mediated pathways [80]. While no direct link exists between OC and succinate dehydrogenase (SDH), both influence cancer cell survival via ROS, metabolism, and epigenetic regulation. Interestingly, OC and SDH deficiency exert opposing epigenetic effects: OC reactivates silenced genes through HDAC/SMYD2 inhibition [133], while SDH loss drives hypermethylation via TET suppression [158]. This interplay highlights the potential for combined therapies targeting both histone modifications and DNA methylation in cancers with metabolic–epigenetic vulnerabilities.

The role of OC in lipid metabolism, an often-overlooked aspect of tumor bioenergetics, presents another promising avenue for investigation. Tumor cells exhibit heightened reliance on fatty acid oxidation (FAO) and lipid biosynthesis, allowing them to sustain membrane synthesis, oncogenic signaling, and metabolic flexibility under hypoxia or nutrient deprivation [159]. Olive oil-derived compounds, particularly oleic acid, have demonstrated lipid-modulating properties, though the precise effects of OC on cancer lipid metabolism remain undefined. Given the essential role of sterol regulatory element-binding protein 1 (SREBP-1) and fatty acid synthase (FASN) in tumor lipogenesis [160], it is plausible that OC may interfere with lipid biosynthetic pathways, thereby depriving tumor cells of essential precursors for proliferation and survival. Equally compelling is the hypothesis that OC may inhibit carnitine palmitoyltransferase 1 (CPT1A), the rate-limiting enzyme in mitochondrial fatty acid oxidation, thereby restricting an alternative fuel source that many therapy-resistant tumors exploit. The possibility that OC impairs lipid homeostasis to induce metabolic stress should be investigated in cancer models that exhibit heightened lipid droplet formation and FAO dependence, such as triple-negative breast cancer, prostate cancer, and pancreatic ductal adenocarcinoma.

To fully elucidate the metabolic effects of OC, future investigations must leverage multi-omic platforms capable of integrating metabolic flux analysis, transcriptomics, and proteomics, such as the study performed by Guitsi et al., although this was in context of cancer [154]. A combination of mass spectrometry-based metabolomics and isotope-labeled tracing experiments would allow for a precise quantification of OC’s impact on glucose metabolism, PPP activity, TCA cycle flux, and lipid anabolism. Functional validation using CRISPR-based knockout models targeting PKM2, G6PD (the rate-limiting enzyme of the PPP), CPT1A, and IDH1 could define whether OC’s metabolic effects are enzyme-specific or mediated through broader signaling pathways. Given the intricate interplay between metabolism and therapy resistance, it will also be crucial to explore synergistic interactions between OC and metabolic inhibitors, such as 2-deoxyglucose (a glycolytic inhibitor), metformin (an OXPHOS disruptor), and FASN inhibitors. Furthermore, in vivo studies employing tumor xenografts and patient-derived organoid models will be essential in determining whether OC exerts a clinically relevant impact on tumor metabolism and therapeutic response.

The emergent landscape of metabolic oncology underscores the need for novel agents capable of targeting tumor bioenergetic plasticity. OC, with its apparent ability to modulate mitochondrial integrity, restrict glucose metabolism, and potentially interfere with lipid and PPP flux, represents an intriguing, albeit underexplored, metabolic disruptor in cancer. Our findings on OC-mediated mitochondrial membrane potential restoration in inflammatory settings lend support to the hypothesis that OC stabilizes mitochondrial function while simultaneously inducing bioenergetic stress in tumor cells. As the field progresses, rigorous mechanistic studies will be required to determine whether OC can be effectively leveraged as a metabolic modulator in precision oncology, with a particular emphasis on its role in targeting the metabolic vulnerabilities of therapy-resistant malignancies.

Section Summary:

This section explores OC’s role in disrupting cancer metabolic reprogramming. OC is hypothesized to impair glycolysis and the pentose PPP, depriving cancer cells of essential resources and enhancing oxidative stress. While its direct impact on TCA cycle/OXPHOS needs further study, OC induces mitochondrial depolarization, contributing to apoptosis. OC may also interfere with lipid metabolism, stressing anabolic pathways. Our findings on OC-mediated mitochondrial membrane potential restoration suggest its potential as a metabolic disruptor. Future multi-omic and in vivo studies are crucial to validate OC’s efficacy in targeting cancer metabolic vulnerabilities.

## 9. Gut Microbiome, Cancer, and Oleocanthal

OC’s disruption of tumor metabolism intersects critically with its gut microbiome modulation—a dual mechanism rooted in its polyphenolic properties. We now examine how OC’s bidirectional microbial interactions influence both gastrointestinal and systemic malignancies.

The gut microbiome plays a pivotal role in maintaining gastrointestinal and systemic health, with dysbiosis—an imbalance in microbial composition—being implicated in local and distant cancer development. Due to its ability to influence immune responses, cancer growth, the effectiveness of immune checkpoint inhibitors, immunotherapy, and the avoidance of its negative effects, gut microbiota play a significant role in the management of cancer [161,162]. The term “immuno-oncology-microbiome” (IOM) refers to the immune-mediated interactions between immune cells, tumors, and microorganisms in the TME [163]. According to the IOM, there are primarily two types of interactions between the host microbiome and tumor: (1) interactions involving gut microbes, which influence the host immune system and affect both local and distant tumor growth and survival; and (2) interactions involving intertumoral microbes, which either reside in the TME or tumor/immune cells to influence tumor progression and anti-tumor immunity [164]. On the other hand, of all illnesses, dysbiosis of the gut microbiota is directly linked to the development of tumors, both in regional gastrointestinal malignancies and other, distant tumors [163]. The multifaceted function of the gut microbiome in cancer prevention, carcinogenesis, and anti-cancer therapy is highlighted by metabolomics and metagenomics research [164].

Polyphenols, have emerged as critical modulators of gut health and microbiota composition, exerting prebiotic, antimicrobial, and anti-inflammatory effects that may have profound implications for cancer prevention and treatment [165]. Numerous polyphenols have demonstrated gut-modulating properties, with specific mechanistic insights emerging from both in vitro and in vivo models [166,167].

**Figure 5:**
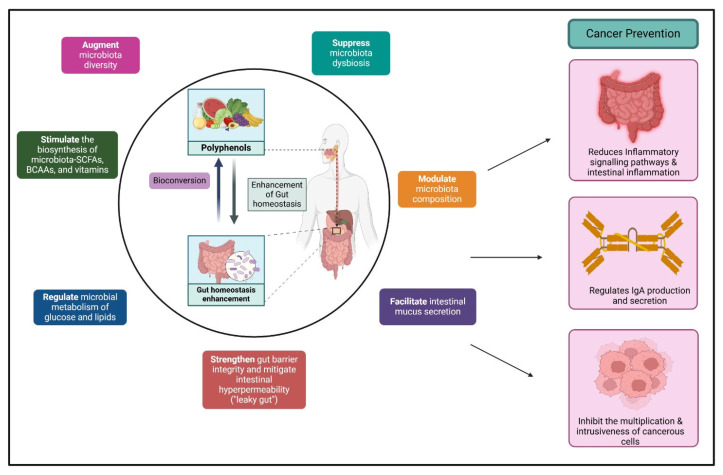
Role of polyphenols in gut microbiota regulation and cancer prevention. This schematic illustrates the beneficial effects of polyphenols on gut microbiota homeostasis and their potential role in cancer prevention. Polyphenols, derived from dietary sources such as fruits and vegetables, undergo bioconversion in the gut, leading to enhanced gut homeostasis. The diagram highlights several key mechanisms through which polyphenols influence microbiota composition, metabolic activity, and gut barrier integrity, ultimately contributing to reduced inflammation, improved immune function, and cancer inhibition.

Polyphenols and gut bacteria have a reciprocal interaction [165]. Although phenolic compounds can positively impact the composition and function of gut microbial populations, gut microbiota is engaged in the bioconversion of polyphenols, modifying their stability, metabolism, and bioactivity [168]. This exemplifies the mutually beneficial interaction between gut bacteria and polyphenols in cancer prevention. Gut microbiota is the key to the biotransformation of dietary polyphenols into bioactive molecules that initiate and modulate molecular and physiological processes involved in the prevention of cancer. On the other side, polyphenols serve as an army for gut homeostasis against pathogens and enhance gut health. In this way, polyphenols may target cancerous cells through the modulation of microbe-derived molecular mechanisms (summarized in Figure 5).

Additionally, polyphenols can have an impact on the intestinal ecology and maintain the balance of the gut’s microbes by acting as both a prebiotic and an antimicrobial agent that prevents pathogenic bacteria like E. coli, Clostridium perfringens, Clostridium histolyticum, and H. pylori from growing and colonizing the body [168]. Furthermore, polyphenols affect the metabolites produced by the microbiota such as SCFA, luminal oxygen levels, mucus production, the intestinal immune system and inflammation, and intestinal integrity. Similarly, bacteria mediate the metabolism of tea polyphenols (TPs), for example, by removing the gallic acid moiety and ring fission to release phenolic acid catabolites, whereas TPs may contribute to the modification of bacterial profiles that enhance gut barrier function, for example, by increasing intestinal permeability to reduce inflammation—reshaping microbial composition—and associated metabolites that exert systemic protection on host metabolism [169].

The gut microbiota can also affect the bioaccessibility and bioavailability of polyphenols through the carbon-to-carbon separation of aromatic and heterocyclic rings in flavonoids, as well as through the dehydroxylation, decarboxylation, and/or hydrogenation of alkene moieties [170]. There is mounting evidence that the gut microbiome plays a critical role in the metabolism and absorption of polyphenols and is thought to be the primary driver of much of the interindividual variation in their bioavailability [171]. The occurrence of metabolic phenotypes in the population can be linked to the various production patterns of polyphenol metabolites obtained by the gut microbiota. Because the gut microbiota substantially metabolizes polyphenols, interindividual variances in bacteria-converting species may play a role in interperson variability.

There is mounting evidence that conventional chemotherapy can considerably benefit from the addition of some natural dietary polyphenols [172,173,174]. One polyphenol whose capacity to prevent BC has been studied is carnosol. In vitro and in vivo investigations have revealed that this chemical dramatically reduces invasion and metastasis. Carnosol reduces matrix metalloproteinase (MMP)-9 activity and the STAT3 signaling pathway by degrading the STAT3 protein in a proteasome-dependent manner to inhibit BC and the STAT3 signaling pathway, respectively [175].

When discussing OC, it is important to keep in mind that protected by the monounsaturated fatty acid triglycerides matrix of EVOO, simple EVOO tyrosol and hydroxytyrosol-based phenolics can be rapidly absorbed in the small intestine, unlike the more complex phenolics, which can reach, at least in part, the large intestine, where they can interact with host gut microbiota (GM) [176]. Consequently, it is important to understand if OC would modulate GM taxonomical distribution and metabotypes independent of systemic OC metabolic fate following absorption. Here, we can also note that absorption of lipid-entrained OC via the chylomicron route is of likely importance, opening the possibility for enterohepatic cycling, which to our knowledge remains to be addressed. In the context of the above, its important to note that in a single-dose safety study of OC, Swiss albino mice tolerated oral doses of up to 500 mg/kg, whereas the intraperitoneal LD50 of OC was estimated to be in the range of 164–524 mg/kg [177]. This disparity clearly shows the great prospective importance of roles played by GM in the safety of OC and EVOO phenolics with oral administration/consumption.

The study [178] identified over 50 bacterial species significantly suppressed by OC across all samples, while 28 species exhibited marked increases. Notably, members of the Bacteroidia class, particularly *Bacteroides fragilis*, have been implicated in colorectal carcinogenesis. Enterotoxigenic *Bacteroides fragilis* (ETBF) produces a toxin that compromises epithelial barrier integrity, induces DNA damage, and fosters a pro-inflammatory microenvironment conducive to tumorigenesis. Its role in CRC is well-documented, given its ability to modulate immune responses and drive chronic inflammation [179,180]. Intriguingly, *Bacteroidia class* was among the most suppressed bacterial species in OC-treated mice, suggesting a potential anti-cancer benefit associated with OC consumption.

Another beneficial gut bacterium modulated by OC in previous studies is *Monoglobus pectinilyticus*, a member of the *Ruminococcaceae* family [181]. *M. pectinilyticus* plays a crucial role in human colonic metabolism as a specialized pectin-degrading bacterium. Its unique glycobiome enables efficient pectin degradation, generating polysaccharide degradation products (PDPs) that serve as substrates for other gut microbes [182]. This metabolic activity is critical for maintaining gut homeostasis and may influence cancer risk by modulating microbial composition and function. While *M. pectinilyticus* itself has not been directly linked to CRC, its role in fostering a balanced gut microbiome and producing anti-inflammatory metabolites could contribute to protective effects against colorectal carcinogenesis.

The gut microbiome plays a pivotal role in CRC pathogenesis, with microbial dysbiosis leading to chronic inflammation, the production of carcinogenic metabolites, and the disruption of the mucosal barrier [183]. By selectively promoting beneficial bacteria while suppressing pro-carcinogenic species, OC may exert a favorable impact on gut microbiota composition, thereby reducing CRC risk.

In contrast, *Prevotella* species demonstrate context-dependent roles in cancer, functioning as either pro-tumorigenic pathobionts or protective commensals depending on microbial community dynamics and host immune interactions. In OC-treated groups, three *Prevotella* strains were notably suppressed. The role of *Prevotella* in CRC remains complex—while some species, such as *Prevotella intermedia*, have been enriched in colorectal adenocarcinoma tissues and are associated with enhanced CRC cell migration and invasion, others may exert protective effects. *P. intermedia* has been implicated in the malignant transformation of colorectal adenomas, particularly in synergy with *Fusobacterium nucleatum*, highlighting its potential contribution to CRC progression and metastasis. Conversely, higher *Prevotella* abundance in the gut microbiota has also been correlated with reduced CRC progression and lower mortality rates, underscoring its multifaceted role in colorectal oncogenesis [184,185].

As mentioned previously OC’s modulation of gut microbiota extends beyond local gastrointestinal effects; in breast cancer, *Prevotella copri* has been identified as significantly enriched in the gut microbiota of breast cancer patients. Mechanistically, *P. copri* promotes breast cancer growth by metabolizing tryptophan, leading to a depletion of indole-3-pyruvic acid (IPyA), a naturally occurring anti-cancer agent. The reduction in IPyA subsequently inactivates the AMPK signaling pathway, facilitating tumor growth [186].

Furthermore, OC’s potential influence on distal tumors through gut–brain and gut–liver axes should be noted. Microbial metabolites modulated by OC—particularly SCFAs and tryptophan derivatives such as kynurenine—directly shape the brain tumor microenvironment by regulating anti-tumor immunity and inflammatory signaling. Additionally, OC’s correction of gut dysbiosis may restore immune balance, counteracting the pro-tumorigenic shift in regulatory and effector immune populations that drives brain cancer progression [187]. Quesada et al. (2023) demonstrated in an ex vivo mouse model that OC intake significantly alters gut microbiota composition, enriching beneficial bacteria linked to SCFA production, such as butyrate producers (*Faecalibacterium* spp.) [178]. This aligns with Farràs et al. (2020), who highlighted that olive oil phenolics like OC elevate SCFAs (e.g., butyrate), which exert anti-inflammatory and anti-tumorigenic effects systemically [176]. The central mechanisms through which butyrate modulates the immune system is by promoting the differentiation and function of regulatory T cells (Tregs), a subset of immune cells known for their role in maintaining immune tolerance [188]. Butyrate influences the epigenetic regulation of Tregs by inhibiting histone deacetylases (HDACs), which enhances the expression of genes essential for Treg development and function [189]. This mechanism helps maintain immune balance while suppressing pro-inflammatory responses that promote tumor-associated inflammation and cancer progression. On the other hand, direct evidence linking OC to kynurenine modulation remains lacking; its suppression of *Prevotella copri* (a tryptophan-metabolizing pathobiont), however, suggests a plausible indirect role in limiting kynurenine-driven neurotoxicity and tumor immune escape, as observed in glioblastoma (GBM)-associated dysbiosis.

Investigations into the gut microbial ecosystem of GBM patients, the most aggressive brain malignancy, have revealed significant dysbiosis. Studies show GBM patients often exhibit higher microbial diversity and bacterial overgrowth, marked by an increase in Proteobacteria (e.g., *Escherichia coli*). Elevated levels of *Enterobacteriaceae*, *Bacteroidaceae* (including *Bacteroides vulgatus*), and *Lachnospiraceae* are observed, alongside notably lower levels of beneficial genera such as *Faecalibacterium* and certain *Prevotella* species [187]. Given OC’s documented ability to enrich beneficial bacteria like *Faecalibacterium* (a key butyrate producer) [178] and suppress problematic groups such as *Bacteroides* and certain *Prevotella* strains, these findings collectively suggest that OC could potentially modulate the profound gut dysbiosis seen in GBM patients. By restoring microbial balance, OC may help counteract the pro-tumorigenic environment influenced by these specific bacterial alterations, opening new avenues for understanding and potentially treating GBM through gut microbiome modulation.

Similarly, OC’s ability to modulate gut dysbiosis presents a promising therapeutic avenue for hepatocellular carcinoma (HCC), a malignancy significantly influenced by the gut–liver axis [190]. HCC pathogenesis is driven by intestinal dysbiosis and compromised gut barrier function, facilitating bacterial translocation and chronic hepatic inflammation. HCC-associated dysbiosis is characterized by reduced microbial α-diversity, depleted commensal genera like *Lactobacillus* and *Bifidobacterium*, and an overrepresentation of pathogenic taxa such as *Enterobacteriaceae*, *Enterococcus*, *Bacteroides*, and *Ruminococcaceae* [191]. OC, through its demonstrated capacity to suppress problematic bacteria (e.g., *Bacteroides*) and foster beneficial ones (e.g., *Monoglobus pectinilyticus* and *Faecalibacterium*), may help restore this crucial balance. Furthermore, OC’s influence on microbial metabolites like SCFAs and bile acids (BAs) is critical. Dysbiosis-driven alterations in BA profiles, particularly the downregulation of FXR, contribute to hepatotoxicity and activate pro-inflammatory signaling in HCC [192,193]. Additionally, exogenous SCFA administration in a hepatitis B virus (HBV)-induced murine model of HCC has shown to significantly reduce the number of dysplastic nodules and tumor burden in HBx-transgenic mice [194]. Corroborating these findings, stool microbiome analyses in HCC patients have revealed a marked depletion of SCFA-producing bacterial genera, such as *Lachnospira*, *Ruminococcus*, and *Butyricicoccus*, suggesting a disrupted SCFA-mediated regulatory axis in human HCC pathophysiology [195]. OC’s role in rebalancing the microbiome could thus help normalize BA metabolism and enhance protective SCFA levels, thereby mitigating chronic inflammation and disrupting carcinogenic pathways in the liver, offering a novel approach to HCC prevention and treatment.

Collectively, these findings highlight the intricate interplay between OC and gut microbiota in the context of cancer. By selectively suppressing pro-tumorigenic bacteria, such as *B. fragilis* and specific *Prevotella* strains, while fostering beneficial microbial populations like *Faecalibacterium* spp. and *M. pectinilyticus*, OC may exert a protective role in cancer prevention. These results underscore the therapeutic potential of OC as a modulator of gut microbiota composition, paving the way for further investigation into its application in cancer treatment and prevention.

Given the growing body of evidence, OC’s interaction with the gut microbiota presents a promising avenue for cancer prevention and treatment. Its ability to modulate bacterial populations, suppress pro-carcinogenic species, and promote gut homeostasis highlights its potential as a natural therapeutic agent. Future research should aim to elucidate the precise molecular mechanisms underlying these effects and explore its clinical applications in oncology.

Section Summary:

This section elucidates OC dual mechanism in cancer modulation: direct effects and critical interactions with the gut microbiome. Dysbiosis is pivotal in cancer development, influencing immunity and therapy. Polyphenols, including OC, reciprocally modulate gut health, acting as prebiotics and antimicrobials, influencing the microbial metabolism of SCFAs, and impacting OC’s systemic fate. Studies show that OC suppresses pro-carcinogenic bacteria like *Bacteroides fragilis* and specific *Prevotella* strains while promoting beneficial ones like *Monoglobus pectinilyticus*. This selective modulation helps reduce cancer risk, notably in colorectal cancer, and extends to modulating breast cancer through *Prevotella copri* and glioblastoma and HCC via the gut–brain and gut–liver axis and SCFA production. Collectively, OC’s therapeutic potential as a gut microbiota modulator for cancer prevention and treatment is underscored.

## 10. Synergistic Effects and Combination Therapies

OC’s pleiotropic mechanism positions it as an ideal candidate for combination therapies across malignancies as summarized in Table 6. This section explores how OC amplifies the efficacy of standard treatments—PARP inhibitors, taxanes, FLT3 inhibitors—while mitigating chemoresistance and toxicity, offering a roadmap for its integration into precision oncology paradigms.

### 10.1. Prostate Cancer: The SMYD2-AR Axis and Its Therapeutic Vulnerabilities

OC’s ability to inhibit SMYD2 has significant implications for prostate cancer treatment, particularly in disrupting the methylation of the androgen receptor (AR) at lysine residues 6–42 [133]. This post-translational modification plays a crucial role in maintaining AR stability and its transcriptional activity. Under normal circumstances, SMYD2-mediated methylation effectively prevents AR ubiquitination and subsequent proteasomal degradation, thereby sustaining oncogenic signaling even in environments with reduced androgen levels, such as those following androgen deprivation therapy [196]. The ability of OC to destabilize AR through SMYD2 inhibition offers multiple therapeutic advantages.

First, by reducing AR stability, OC could enhance the efficacy of AR antagonists such as enzalutamide and apalutamide, both of which function by preventing AR nuclear translocation [197]. However, resistance to these drugs frequently arises due to the emergence of AR splice variants such as AR-V7, which remain active despite androgen blockade [198]. The inhibition of SMYD2 may play a role in decreasing the stability of AR-V7, thereby potentially delaying or even preventing the development of resistance to these therapies.

Additionally, there is a growing body of evidence suggesting that AR signaling is involved in upregulating the expression of DNA repair genes [199,200,201]. Consequently, the degradation of AR induced by OC could impair homologous recombination repair mechanisms, thereby sensitizing prostate cancer cells to the effects of PARP inhibitors such as olaparib. This presents a potential synergistic therapeutic strategy for patients with BRCA-proficient tumors.

Furthermore, the OC-mediated suppression of AR could enhance the efficacy of taxane-based chemotherapy. For instance, docetaxel exerts its anti-cancer effects by hyperstabilizing microtubules and inducing mitotic arrest [202]. However, AR signaling has been implicated in promoting cellular survival through the upregulation of anti-apoptotic proteins such as Bcl-2 [203]. By downregulating AR expression, OC may amplify the pro-apoptotic effects of taxane chemotherapy, thereby increasing the likelihood of tumor cell death.

To validate these hypotheses, preclinical testing could involve the use of LNCaP/AR+ xenograft models to assess the kinetics of AR degradation following OC treatment. Additionally, combining OC with enzalutamide in these models while monitoring PSA levels and metastatic progression through bioluminescent imaging would provide further insights into its therapeutic potential.

### 10.2. Pancreatic Ductal Adenocarcinoma: The Role of EMT Suppression, Autophagy Modulation, and Bacterial Coinfection

In pancreatic ductal adenocarcinoma (PDAC), OC has demonstrated the capacity to suppress EMT by downregulating the Snail/Slug-Twist axis and restoring the expression of E-cadherin [122]. Given that EMT is a key driver of metastasis in pancreatic cancer, this mechanism could significantly reduce the tumor’s ability to invade and disseminate [204]. This effect is particularly relevant in combination with existing therapeutic regimens.

For example, the standard-of-care FOLFIRINOX chemotherapy regimen induces cytotoxic stress, which paradoxically triggers EMT and promotes cancer cell dissemination [205]. By inhibiting EMT, OC may help counteract this undesired effect, thereby potentially enhancing the long-term efficacy of FOLFIRINOX.

Another significant mechanism by which OC may exert its anti-cancer effects is through the modulation of autophagy—a mechanism inferred from studies on other polyphenols [75]. Autophagy plays a pivotal role in sustaining metabolic homeostasis in cancer cells, especially in the setting of therapeutic resistance. Notably, oleuropein has been shown to inhibit autophagosome formation, as demonstrated by the suppression of LC3-II expression. This inhibition may compromise the capacity of gemcitabine-resistant cancer cells to recycle nutrients and maintain survival under stress conditions [206]. It is plausible that OC could exert a similar inhibitory effect on autophagy. This property could be therapeutically leveraged by combining OC with oxidative stress-inducing agents such as gentamicin. In autophagy-compromised cells, the accumulation of ROS induced by such agents may result in enhanced cell death.

In addition to its direct effects on tumor biology, OC may also modulate the tumor microenvironment, particularly in the context of bacterial coinfection. Notably, Klebsiella pneumoniae infections have been identified as drivers of EMT in PDAC, further exacerbating the tumor’s invasive potential [207]. OC’s well-documented anti-inflammatory properties may counteract infection-driven metastasis while simultaneously enhancing the bactericidal effects of gentamicin.

Preclinical models that could be used to evaluate these effects include KRASG12D organoids, which can be treated with OC in combination with MEK inhibitors such as trametinib to assess its impact on RAS pathway addiction. Additionally, orthotopic pancreatic cancer models subjected to lipopolysaccharide (LPS) challenge can be employed to mimic infection-induced inflammation and evaluate OC’s ability to suppress tumor progression in an inflammatory microenvironment.

### 10.3. Glioblastoma Multiforme: Blood–Brain Barrier Penetration and Overcoming TMZ Resistance

Despite the challenges associated with blood–brain barrier (BBB) penetration, OC’s relatively low molecular weight (304 Da), moderate lipophilicity (logP ~2.5), and structural analogs (e.g., oleuropein), showing central nervous system (CNS) activity [208], suggest that it may possess some degree of CNS bioavailability [59]. If OC is capable of crossing the BBB, its potent anti-inflammatory effects could be leveraged to inhibit key signaling pathways involved in glioblastoma progression. In particular, NF-κB and COX-2 suppression could mitigate microglia-driven IL-6/STAT3 signaling, which is known to contribute to the maintenance of glioblastoma stemness [209].

Temozolomide (TMZ), the frontline chemotherapeutic agent for glioblastoma, frequently encounters resistance due to the upregulation of O6-methylguanine-DNA methyltransferase (MGMT), an enzyme that counteracts the alkylating effects of TMZ. Interestingly, TNF-α signaling has been implicated in the regulation of MGMT expression [210], and given that OC has been shown to inhibit TNF-α, it is conceivable that it could enhance TMZ sensitivity by reducing MGMT levels.

To test this hypothesis, brain organoid models co-cultured with glioblastoma stem cells and neurons/glia could be employed to evaluate OC’s impact on tumor–niche interactions. Furthermore, the use of CRISPR-edited glioblastoma organoids with IDH1 mutations may provide additional insights into potential metabolic synergies between OC and current therapeutic strategies.

### 10.4. Hematological Malignancies: Mitochondrial Priming and Topoisomerase Interplay

In multiple myeloma (MM), OC exerts its effects by inhibiting MIP-1α/CCL3 signaling, thereby disrupting the adhesion of MM cells to stromal components and reducing IL-6 production [211]. These properties suggest potential synergy with existing MM therapies, including venetoclax and carfilzomib. By downregulating Bcl-2, OC lowers the mitochondrial threshold for apoptosis, enhancing the efficacy of venetoclax. Furthermore, its ability to activate ERK and JNK pathways amplifies proteotoxic stress, potentially augmenting the cytotoxic effects of the proteasome inhibitor carfilzomib.

In acute myeloid leukemia (AML), OC may enhance responses to FLT3 inhibitors such as gilteritinib by suppressing STAT3-mediated survival signals in FLT3-ITD mutant cells. Additionally, its capacity to generate ROS via NADPH oxidase activation may exacerbate the DNA strand breaks induced by topoisomerase inhibitors like etoposide [212].

For experimental validation, MM co-cultures with HS-5 stromal cells could be utilized to mimic the protective bone marrow microenvironment. Similarly, AML patient-derived xenograft (PDX) models with TP53 mutations could be used to evaluate OC’s efficacy in chemotherapy-resistant settings.

### 10.5. Chemotoxicity Mitigation and Bcl-2 Inhibitor Synergy

OC exhibits a dual role in redox modulation, exerting pro-oxidant effects in cancer cells while displaying antioxidant properties in normal tissues, as discussed previously. In normal cells, OC upregulates Nrf2/HO-1 signaling, offering protective effects against cisplatin-induced nephrotoxicity and doxorubicin-mediated cardiotoxicity [129,213]. In preclinical models, the co-administration of OCs structural analogs with cisplatin has been shown to reduce nephrotoxicity while simultaneously increasing tumor apoptosis through p53 stabilization [214].

Furthermore, a study by Carter et al. demonstrated that the inhibition of MCL-1 sensitizes AML cells and AML stem/progenitor cells to BCL-2 inhibition, including those with intrinsic and acquired resistance to venetoclax. This effect was mediated through the cooperative release of pro-apoptotic proteins BIM, BAX, and BAK from anti-apoptotic BCL-2 proteins, as well as the suppression of cell metabolism and key stromal microenvironmental mechanisms. The combined inhibition of MCL-1 (using the MCL-1 inhibitor AZD5991) and BCL-2 (using venetoclax) significantly extended survival in mice bearing patient-derived xenografts from an AML patient who had developed resistance to venetoclax/decitabine. These findings highlight that the dual targeting of MCL-1 and BCL-2 enhances therapeutic efficacy and overcomes both intrinsic and acquired resistance to BCL-2 inhibition [215].

Given that OC has been shown to inhibit MCL-1 [137]—a key resistance factor in venetoclax-treated MM and AML [216]—it is reasonable to anticipate potential synergism between OC and BCL-2 inhibitors such as venetoclax. This combination may similarly enhance apoptotic susceptibility and overcome resistance mechanisms observed in hematological malignancies.

To investigate these effects, dynamic BH3 profiling could be employed to measure mitochondrial priming in primary MM and AML cells following OC exposure. Additionally, 3D bone marrow mimetic scaffolds could be utilized to evaluate stroma-mediated resistance to venetoclax in combination with OC.

OC’s multimodal impact across different malignancies, particularly in disrupting oncogenic pathways, modulating inflammation, and enhancing chemosensitivity, underscores its promise as a potential adjunctive therapeutic agent. Further preclinical and clinical investigations are however warranted to fully elucidate its translational potential and optimal combinatorial strategies when it comes to precision oncology.

Section Summary:

This section underscores OC’s potential as a versatile agent for combination therapies due to its diverse mechanisms. In prostate cancer, OC’s SMYD2 inhibition can destabilize AR, potentially enhancing AR antagonists, PARP inhibitors, and taxanes. For PDAC, OC may suppress EMT and modulate autophagy, improving chemotherapy efficacy and counteracting bacterial coinfection. In glioblastoma, OC could enhance temozolomide sensitivity by inhibiting TNF-α. In hematological malignancies, OC may enhance FLT3 and Bcl-2 inhibitor responses by disrupting survival signals and inhibiting MCL-1. Additionally, OC exhibits a dual role in chemotherapy mitigation, protecting normal tissues while sensitizing cancer cells. These multifaceted actions position OC as a promising adjunct to standard cancer treatments, warranting further clinical investigation.

## 11. Future Directions, Therapeutic Potential, and Critical Research Priorities

### 11.1. Translating PAR-2 Modulation by Oleocanthal into Chondrosarcoma Therapeutics

Our preliminary findings demonstrating the ability of OC to downregulate PAR-2 expression in both transcriptomic and proteomic assays warrant exploration of its therapeutic applicability beyond inflammatory cartilage pathologies. Specifically, there is a compelling mechanistic rationale to extend this PAR-2 modulatory effect to chondrosarcoma (CHS), a malignant cartilage tumor characterized by profound ECM remodeling, immune evasion, and stemness-associated dedifferentiation. Emerging evidence identifies PAR-2 as a critical mediator of inflammation-driven cartilage degeneration, and recent work has confirmed OC’s capacity to suppress PAR-2 expression alongside downstream inflammatory cascades in osteoarthritic chondrocytes [150]. Given the chondrocytic origin shared by osteoarthritis (OA) and CHS, and the central role of PAR-2 in tumorigenic inflammation and matrix degradation, this mechanistic axis represents a promising therapeutic target in CHS.

A priority research direction involves elucidating the role of PAR-2/NF-κB crosstalk in regulating CHS stemness and progression. CHS is frequently driven by IDH1/2 mutations that induce global DNA hypermethylation and promote SOX9-mediated dedifferentiation programs [134]. It is hypothesized that PAR-2 activation in CHS may stabilize β-catenin through calcium-dependent disheveled (DVL) phosphorylation, amplifying Wnt/β-catenin signaling to sustain cancer stem cell populations. Future studies should investigate whether OC disrupts this PAR-2/RAC1/β-catenin axis in IDH-mutant CHS models, leveraging its dual capacity to downregulate PAR-2 [150] and preserve repressive H3K27me3 epigenetic marks [132] to counteract CpG island hypermethylation at tumor suppressor loci such as CDKN2A.

Additionally, OC’s previously demonstrated ability to stabilize collagen type II alpha 1 chain (COL2A1) expression in OA chondrocytes [150] may hold relevance in CHS, where ECM remodeling and collagen mutations create an immunosuppressive tumor niche enriched in M2 macrophages [134]. Notably, COL2A1 truncation mutations (e.g., p.Gly1170Val) in CHS impair matrix integrity and immune infiltration. OC-mediated restoration of COL2A1 expression may counteract this process, thereby enhancing CD8^+^ T cell infiltration. Parallel investigations should focus on evaluating OC’s impact on PAR-2-driven secretion of chemokines such as CCL2/MCP-1, which contribute to tumor-associated macrophage (TAM) recruitment and immune evasion. Single-cell RNA sequencing of OC-treated CHS xenografts would provide high-resolution insight into the modulation of tumor–immune interactions.

From a metabolic perspective, IDH1/2 mutations in CHS promote the accumulation of the oncometabolite 2-hydroxyglutarate (2-HG), impairing mitochondrial electron transport chain (ETC) activity and contributing to cellular dedifferentiation [30]. Our preliminary data demonstrating OC’s capacity to preserve mitochondrial membrane potential (ΔΨm) in LPS-stimulated chondrocytes [150] suggest a possible metabolic synergy between OC and IDH-targeted therapies (e.g., AG-120). Future studies employing CHS organoid models should assess whether OC-mediated restoration of ETC function and suppression of PAR-2/IL-1β-driven glycolytic reprogramming enhances the efficacy of IDH inhibitors and sensitizes tumors to DNA repair inhibitors, such as PARP inhibitors, through the attenuation of 2-HG-induced DNA repair defects (e.g., FANCD2 mono-ubiquitination).

Furthermore, CHS progression is associated with STAT3 hyperactivation via IL-6/JAK2 signaling, which drives dedifferentiation through SOX4-mediated transcriptional programs [134]. Given prior evidence of OC’s capacity to inhibit PAR-2-dependent STAT3 phosphorylation at Ser727 in OA models [217], it is plausible that OC may disrupt this pro-tumorigenic STAT3–PAR-2 feedforward loop in CHS. Experimental validation employing CRISPR-Cas9-mediated PAR-2 knockout in established CHS cell lines (SW1353, JJ012) will be instrumental in delineating whether OC’s anti-tumorigenic effects extend beyond PAR-2-dependent mechanisms.

One of the principal challenges in CHS therapy is the dense collagenous stroma, which impedes effective drug penetration and contributes to chemoresistance. OC’s amphiphilic structure lends itself to nanotherapeutic encapsulation strategies. Specifically, lipid–polymer hybrid nanoparticles (LPHNs) functionalized with CHS-specific surface ligands (e.g., anti-CD44v6) could be engineered for OC delivery, facilitating tumor-specific uptake and stromal penetration. Preclinical validation should assess whether such OC-LPHNs improve doxorubicin accumulation in CHS xenografts while simultaneously attenuating PAR-2/RANKL-driven osteolytic activity [150].

To operationalize these strategies, we propose a translational roadmap comprising three phases:

*Phase 1*: Validation of OC-mediated PAR-2 suppression and downstream transcriptomic alterations in IDH1-R132C mutant CHS patient-derived organoids, employing multiplexed Nanostring PanCancer Pathways panels.

*Phase 2*: Evaluation of the combinatorial efficacy of OC with demethylating agents such as decitabine (DAC) to exploit potential epigenetic synergy—specifically, DAC’s capacity to reverse IDH-mediated DNA hypermethylation coupled with OC’s stabilization of H3K27me3 at oncogenic loci [132].

*Phase 3*: Development of non-invasive imaging modalities through the synthesis of PAR-2/COL2A1 dual-targeted positron emission tomography (PET) tracers (e.g., ^68^Ga-OC-DOTA conjugates) to monitor OC’s therapeutic efficacy and ECM remodeling capacity in vivo.

Collectively, these future investigations will clarify the therapeutic relevance of PAR-2 suppression by OC in chondrosarcoma and establish a framework for the clinical translation of OC-based multimodal therapies targeting tumor inflammation, stemness, metabolism, and the immunosuppressive microenvironment.

### 11.2. Translating Oleocanthal’s PAR-2/TNF-α/Calcium Signaling Modulation into Colorectal Cancer Therapeutics

Our preliminary investigations demonstrating OC’s ability to downregulate PAR-2 and TNF-α expression in CRC models [83] provide a strong mechanistic rationale to further explore its therapeutic potential in modulating key oncogenic signaling cascades. Given the established role of PAR-2-mediated calcium flux and TNF-α-induced inflammation in sustaining CRC progression, EMT, and immune evasion, we propose a comprehensive translational strategy to leverage OC’s multimodal actions.

A principal research priority is to delineate OC’s capacity to disrupt the PAR-2–TNF-α–ERK1/2–TROP-2 axis, which orchestrates metastatic dissemination in CRC. Building upon our findings, systematic in vitro studies should evaluate OC’s dose-dependent inhibition (20–150 µg/mL) of TROP-2 expression and ERK1/2 phosphorylation in HT-29 and Caco-2 cell lines under TNF-α stimulation (5–20 ng/mL). Functional validation through TROP-2 knockdown using siRNA in combination with OC treatment should be performed, assessing resultant effects on cell invasion (Transwell assays) and matrix degradation (MMP-2 and MMP-9 activity by zymography) [218]. Further mechanistic depth should be achieved by pharmacologically inhibiting ERK1/2 (e.g., SCH772984) to establish whether OC’s anti-invasive effects are ERK-dependent.

Additionally, OC’s modulation of calcium signaling in CRC cells [83] merits detailed evaluation. Calcium flux serves as a critical mediator of Wnt/β-catenin and NFAT pathway activation, both of which are central to CRC progression. Future studies should employ live-cell calcium imaging using Fluo-4 AM to characterize OC-induced alterations in intracellular calcium dynamics during EMT in CRC spheroids. Parallel assays should assess OC’s effects on β-catenin nuclear translocation (via immunofluorescence) and LEF/TCF transcriptional activity, particularly under conditions of ER calcium depletion induced by thapsigargin. Translationally, combination studies evaluating OC with calcium channel blockers (e.g., verapamil) in APC-mutant patient-derived organoids (PDOs) should be prioritized to target store-operated calcium entry (SOCE)-driven proliferative signaling.

OC’s regulatory effects on the TME also warrant investigation. Given that TNF-α signaling amplifies IL-6 and IL-8 secretion in the CRC TME, fostering apt conditions for tumor invasion [219], it is imperative to evaluate OC’s capacity to attenuate this cytokine axis. Co-culture models using HT-29 cells and THP-1-derived macrophages can be employed to assess the dose-dependent suppression of IL-6 and IL-8 (via multiplex Luminex assays) alongside flow cytometric profiling of M2 macrophage markers (CD206^+^). Spatial transcriptomic analyses of OC-treated CRC xenografts should further resolve the compartmental localization of PAR-2, TNF-α, and cytokine expression within stromal and epithelial niches. Correlative studies linking OC-induced cytokine shifts with PD-L1 expression and CD8^+^ T cell infiltration across microsatellite-stable (MSS) and microsatellite instability-high (MSI-H) CRC subtypes will offer insights into OC’s immunomodulatory potential.

A critical area for mechanistic exploration is the role of PAR-2–calcium crosstalk in sustaining CRC stemness. PAR-2 activation is known to maintain LGR5^+^ CRC stem cells via calcium-dependent YAP/TAZ signaling. It is therefore pertinent to assess whether OC disrupts this signaling axis by treating primary CRC stem cells (CD133^+^/CD44^+^) with OC in the presence or absence of the PAR-2 agonist SLIGRL-NH_2_, followed by an evaluation of stemness markers (OCT4, NANOG) and organoid-forming capacity. Real-time imaging of calcium oscillations in stem-like niches can be performed using genetically encoded FRET-based calcium biosensors (GCaMP6s) to visualize OC’s inhibitory effects. Furthermore, combinatorial studies with Wnt pathway inhibitors (e.g., LGK974) in RSPO fusion-positive PDOs may offer a synergistic therapeutic strategy.

Beyond these signaling effects, OC’s reported ability to preserve H3K27me3 marks [83] opens avenues for epigenetic intervention. Chromatin immunoprecipitation sequencing (ChIP-seq) should be utilized to assess H3K27ac and H3K27me3 enrichment at PAR-2 (F2RL1) and TNF-α promoter loci following OC treatment. Complementary CRISPR-dCas9-KRAB-mediated silencing of PAR-2, combined with OC administration, can clarify additive effects on TNF-α suppression (quantified via ELISA). Additionally, DNA methylation profiling using the Illumina EPIC array will help identify epigenetic biomarkers predictive of OC responsiveness in CRC PDOs.

Given the physical and biochemical barriers of CRC desmoplasia that limit OC bioavailability, nanotherapeutic strategies are warranted. The development of fibroblast activation protein (FAP)-targeted liposomes encapsulating OC (L-OC) is proposed, leveraging FAP’s overexpression in CRC CAFs [83]. L-OC penetration and biodistribution should be assessed in decellularized CRC extracellular matrix scaffolds using mass spectrometry imaging and orthotopic patient-derived xenograft (PDX) models. For theranostic applications, the radiolabeling of L-OC with ^68^Ga-DOTA will enable the PET/CT monitoring of PAR-2–rich metastatic lesions.

Finally, OC’s potential to overcome therapeutic resistance through synergy with TNF-α–ERK1/2-directed therapies should be investigated. Preclinical screening should evaluate the combinatorial efficacy of OC with ERK1/2 inhibitors (e.g., ulixertinib) in Consensus Molecular Subtype 4 (CMS4) CRC models characterized by high TNF-α expression. Single-cell RNA sequencing (scRNA-seq) of circulating tumor cells (CTCs) treated with OC should map shifts in the TNF-α–NF-κB–IL-8 axis, elucidating OC’s capacity to mitigate pro-metastatic signaling trajectories.

To operationalize these strategies, we again propose a translational roadmap comprising three phases:

*Phase 1:* Validate OC’s inhibition of the PAR-2/TNF-α/calcium signaling triad in APC/KRAS-mutant PDOs (*n* = 50), employing high-content imaging platforms (e.g., Operetta CLS).

*Phase 2:* Conduct preclinical combinatorial trials evaluating OC in conjunction with SOCE inhibitors (e.g., CM2489) in CRC liver metastasis PDX models.

*Phase 3:* Develop OC-loaded hydrogel-based delivery systems for locoregional application in murine models of CRC-associated peritoneal carcinomatosis, assessing therapeutic efficacy and stromal remodeling.

These future directions will comprehensively delineate the mechanistic underpinnings of OC’s multimodal action in CRC and lay the foundation for the rational development of OC-based adjunctive therapies targeting key inflammatory and metabolic vulnerabilities in colorectal carcinogenesis.

### 11.3. Advancing Oleocanthal’s Clinical Potential

To realize OC’s clinical potential, several research priorities will need to be addressed. Systematic pharmacokinetic–pharmacodynamic (PK-PD) modeling comparing various delivery routes (oral, IV, and nanoparticle-mediated) in murine models will be essential to quantify biodistribution and tumor accumulation. Dual-loaded systems combining OC with chemotherapeutics (e.g., paclitaxel) in nanocarriers should be explored to synergize lysosomal disruption (OC) and microtubule inhibition (paclitaxel).

Comprehensive toxicology profiles will assess OC’s effects on normal lysosomes, particularly in renal and hepatic tissues. Scaling up liposome/nanoparticle production under GMP standards will be critical for translational trials. Future studies will prioritize in vivo PK-PD correlations, scalable nanomanufacturing, and combinatorial approaches with immunotherapies.

The integration of advanced delivery systems with molecular targeting strategies will hold transformative potential for enhancing OC’s anti-cancer efficacy. By addressing these challenges, OC could transition from a dietary chemopreventive agent to a cornerstone of precision oncology.

While the preclinical evidence for OC’s anti-cancer potential is robust and multifaceted, clinical validation remains conspicuously limited. To date, a small number of registered trials—such as NCT03528603 and NCT02902913—have assessed oleocanthal-rich EVOO primarily in the context of platelet reactivity and metabolic modulation, while others (e.g., NCT04215367) have explored its effects in chronic lymphocytic leukemia as part of broader dietary interventions. Importantly, none of these studies isolate OC as a therapeutic agent or rigorously evaluate its role in oncology-specific outcomes. This paucity of clinical trials underscores a critical translational gap and reinforces the need for dedicated, well-powered interventional studies that assess OC’s safety, pharmacokinetics, and therapeutic efficacy in cancer. Addressing this unmet need will be pivotal in transitioning OC from a nutraceutical of dietary interest to a viable adjunctive agent in precision oncology.

### 11.4. Strategy for Investigating Protein Expression in Cancer Cells Using Proteomics and Oleocanthal

Future research should employ a multi-tiered experimental approach, integrating advanced mass spectrometry-based proteomics, bioinformatics, and functional validation assays to elucidate OC’s molecular effects on tumor progression and signaling pathways. The *first phase* will involve selecting clinically relevant cancer cell lines, including colorectal (HCT116, SW620, LS174T), breast (MCF-7, MDA-MB-231), liver (HepG2, Huh7), lung (A549, H460), and prostate (PC-3, LNCaP) cancer models, alongside normal epithelial controls for differential analysis. These cells will be maintained under standard culture conditions (RPMI/DMEM + 10% FBS + 1% Pen/Strep) and exposed to increasing OC concentrations (0, 5, 10, 25, 50 µM) over 24–72 h to establish a dose–response curve for proteomic interrogation.

Upon OC treatment, cells will undergo protein extraction using RIPA or urea-based lysis buffers to maximize the solubilization of cytoplasmic, nuclear, and membrane-bound proteins. Protein quantification via BCA or Bradford assay will precede in-solution tryptic digestion, incorporating reduction (DTT), alkylation (IAA), and enzymatic digestion (trypsin or Lys-C) to generate peptides suitable for high-resolution LC-MS/MS analysis. Global proteomic profiling will be conducted using label-free quantification (LFQ) on an Orbitrap-based mass spectrometer or via TMT/iTRAQ-based multiplexed proteomics for comparative quantification across treatment conditions. Data acquisition in a data-dependent acquisition (DDA) or data-independent acquisition (DIA) mode will maximize peptide coverage and enable accurate protein quantitation.

In the *second phase*, targeted proteomics strategies such as Selected Reaction Monitoring (SRM) and Parallel Reaction Monitoring (PRM) should validate the differential expression of proteins associated with key oncogenic pathways, including NF-κB, PI3K/AKT, MAPK, apoptosis regulation (Bcl-2, Bax, Caspase-3, PARP-1), and EMT markers. Post-translational modification (PTM) profiling will assess changes in phosphoproteomics (using TiO2 or IMAC enrichment), acetylation, ubiquitination, and glycosylation following OC treatment. This step is crucial for delineating OC’s mechanistic impact at a post-translational regulatory level, particularly in relation to PAR-2 signaling in inflammation-associated tumorigenesis.

In the *third phase*, validation assays such as Western blotting (WB) for pathway-specific protein analysis, qRT-PCR for transcriptomic correlation, immunoprecipitation (IP) for protein–protein interaction studies, and immunofluorescence (IF) coupled with confocal microscopy to assess subcellular localization of key differentially expressed proteins (DEPs) should be conducted. This phase will also entail the dissemination of functional assays, including MTT/BrdU cell proliferation assays, Annexin V/PI apoptosis detection by flow cytometry, wound healing and transwell migration assays, colony formation efficiency studies, and ROS detection via DCFDA fluorescence, to establish the phenotypic consequences of OC-induced proteomic alterations. Parallelly, high-content imaging and live-cell tracking will monitor real-time cellular responses to OC treatment.

*In the fourth phase*, data processing and pathway enrichment analyses will be conducted using MaxQuant, Perseus, and Scaffold for proteomic data normalization and visualization, followed by Ingenuity Pathway Analysis (IPA), DAVID, and KEGG enrichment mapping to identify statistically significant perturbations in key oncogenic networks. STRING-based protein–protein interaction (PPI) mapping will infer molecular connectivity and predict novel OC-modulated targets.

Furthermore, to ensure translational relevance, patient-derived organoid models should be utilized for further validation, and CRISPR/Cas9-mediated knockout studies targeting OC-regulated proteins will ascertain their functional necessity in oncogenesis. Finally, in vivo validation in xenograft mouse models should be conducted to determine whether OC-mediated proteomic alterations translate into tumor growth inhibition and metastasis suppression.

This integrative strategy will bridge MS-based proteomics with bioinformatics, functional validation, and translational oncology, positioning OC as a promising modulator of oncogenic signaling pathways and a potential therapeutic candidate in cancer research.

### 11.5. AI-Driven Oleocanthal Target Identification in Cancer Therapeutics

Future research should harness artificial intelligence (AI) for OC target identification in oncology through a computationally intensive, multimodal framework integrating deep learning architectures, high-dimensional multi-omics data processing, and predictive pharmacogenomics. AI-driven methodologies will leverage transformer-based models (e.g., BioBERT, GPT-4Bio) for literature mining, convolutional neural networks (CNNs) for structural protein–ligand interaction analysis, and graph neural networks (GNNs) for dynamic protein–protein interaction (PPI) modeling, facilitating the precise identification of OC-regulated molecular targets.

High-throughput proteomic mass spectrometry data (LC-MS/MS) will be processed using Fourier transform-based spectral deconvolution algorithms coupled with machine learning-based feature engineering (e.g., random forest, XGBoost) to extract differentially expressed proteins (DEPs) following OC treatment. Tandem affinity purification (TAP) and cross-linking mass spectrometry (XL-MS) datasets will be subjected to unsupervised clustering models (e.g., hierarchical clustering, t-SNE, UMAP) to establish OC-driven interaction perturbations within the PPI landscape. Deep variational autoencoders (VAEs) will facilitate dimensionality reduction in transcriptomic and epigenomic datasets, enabling the extraction of latent biological signals correlating OC exposure with oncogenic pathway suppression.

Molecular docking and dynamic simulation pipelines will employ hybrid physics-informed neural networks (PINNs) and quantum mechanics/molecular mechanics (QM/MM) simulations to predict OC’s binding kinetics with potential target proteins. AlphaFold2-based protein structure predictions will be integrated with generative adversarial networks (GANs) for ligand-based virtual screening, refining OC-protein docking precision. AI-enhanced binding free energy calculations (MM/PBSA and MM/GBSA models) will inform target validation, ranking protein targets based on predicted OC-binding thermodynamics.

Federated learning models will analyze heterogeneous datasets from multi-omics repositories (e.g., TCGA, CPTAC, LINCS L1000, PRIDE), training meta-classifiers that predict OC efficacy across diverse cancer subtypes. Multi-omics Bayesian networks will uncover causative OC-driven molecular rewiring by modeling perturbation–response trajectories. Causal inference frameworks (e.g., DoWhy, CausalML) will differentiate direct OC-mediated target effects from secondary adaptive responses, ensuring precise mechanistic delineation.

Post hoc interpretability techniques such as SHapley Additive exPlanations (SHAP), Integrated Gradients, and attention-based saliency maps will validate AI-derived target prioritization, ensuring alignment with experimental pharmacodynamics. This integrative AI-driven pipeline will advance systems pharmacology by refining OC’s therapeutic target identification, enabling rational drug repurposing and the development of OC-based precision oncology therapeutics with enhanced efficacy and translational relevance.

### 11.6. Computational Roadmap for Target Identification and Binding Characterization of Oleocanthal

While previous studies have primarily focused on assessing OC’s effects on downstream molecular markers in various cancer cell lines, direct investigations into its interactions with specific receptors or target molecules remain unexplored. Although research such as that by Cusimano et al. has demonstrated OC-induced apoptosis through markers like PARP cleavage, caspase activation, and γH2AX expression in liver (HepG2, Huh7, Hep3B, PLC/PRF/5) and colon (HT29, SW480) cancer cell lines, the precise molecular interactions between OC and its potential targets have not been characterized.

Recognizing this gap, our lab recently explored the direct interaction between OC and PAR2 in CRC cell lines (Caco-2, HT-29). Observing OC-induced PAR2 downregulation through Western blotting and RT-PCR, we hypothesized a potential direct interaction, which was later validated in silico (readers are referred to the article by Patnaik et al. for specific details [83]).

Building upon the preliminary evidence of OC’s interaction with PAR-2, future investigations should adopt an integrative, scalable, and translational computational–experimental strategy to delineate the direct molecular interactome of OC across multiple oncogenic targets. This approach will not only validate observed downstream signaling perturbations but will also facilitate rational design of OC-based therapeutic interventions.

The proposed strategy initiates with sequence-based structural modeling. High-confidence amino acid sequences of candidate OC targets will be retrieved from the UniProt database and subjected to structure prediction using AlphaFold v2.3 or later versions. Structural modeling will be performed using MMseqs2-based multiple sequence alignment against UniRef90, MGnify, and PDB70 datasets, yielding five structural models per target ranked by confidence metrics, including pLDDT scores, predicted aligned error (PAE) matrices, and per-residue pIDDT scores. Transmembrane regions and structured domains with pLDDT > 90 will be prioritized for downstream analysis, while flexible loop regions (pLDDT < 50) will be earmarked for further refinement using Rosetta Membrane protocols or cryo-EM-guided model fitting to enhance atomic-level resolution.

Once structural models are validated, molecular docking analyses will be performed using both blind and focused docking strategies. Platforms such as CB-Dock2, AutoDock Vina (v1.2.0), and GNINA (a deep learning–augmented docking engine) will be employed to predict potential OC binding pockets and binding poses. Ligand preparation will incorporate geometry optimization and energy minimization using the OMEGA toolkit, followed by conversion to PDBQT format for docking studies. Binding poses will be ranked based on binding free energy (ΔG), ligand efficiency indices, and cavity-based scoring functions (PC-score).

For the highest-scoring binding modes, structural and energetic stability will be evaluated using all-atom molecular dynamics (Mol D) simulations in explicit solvent environments. Mol D simulations will be performed using GROMACS (v2023 or later) with CHARMM36m or AMBER ff19SB force fields, spanning simulation trajectories of 200–500 ns. Parameters such as root mean square deviation (RMSD), root mean square fluctuation (RMSF), and hydrogen bond occupancy will be analyzed to assess complex stability. Free energy calculations, including Molecular Mechanics/Poisson–Boltzmann Surface Area (MM/PBSA) or Free Energy Perturbation (FEP) methods, will be employed to quantify the thermodynamic favorability of OC–target interactions.

To ensure translational validity, in silico predictions will be experimentally verified using the site-directed mutagenesis of key interacting residues, followed by binding affinity measurements through surface plasmon resonance (SPR), microscale thermophoresis (MST), or biolayer interferometry (BLI). In parallel, downstream functional assays, such as reporter gene assays, Western blotting, and cell viability studies, will corroborate the biological relevance of identified interactions.

Critically, this computational pipeline is designed to be modular and expandable, enabling the systematic interrogation of OC’s binding interactions beyond PAR-2 to include other validated oncogenic targets such as receptor tyrosine kinases (e.g., c-Met, EGFR), epigenetic modulators (e.g., histone acetyltransferases), and multidrug resistance transporters (e.g., ABCB1, ABCG2). Complementary cheminformatics tools, such as SwissTargetPrediction and the Similarity Ensemble Approach (SEA), may further assist in target identification and prioritization. This integrative strategy will facilitate the construction of a comprehensive OC–target interaction atlas, laying the groundwork for the rational development of OC-derived multi-targeted anti-cancer therapeutics.

Section Summary:

This section outlines OC’s significant therapeutic potential and critical research priorities for its clinical translation, emphasizing combination therapies. Future efforts will focus on translating OC’s PAR-2 modulation for chondrosarcoma and its PAR-2/TNF-α/calcium signaling effects for colorectal cancer, both involving detailed mechanistic studies and nanotherapeutic delivery. Broader advancements require comprehensive PK-PD modeling, toxicology, and scalable nanomanufacturing for clinical trials. Crucially, AI-driven target identification and detailed computational roadmaps will define OC’s precise molecular interactome. Despite robust preclinical data, limited clinical validation underscores the urgent need for dedicated interventional studies to transition OC from a dietary compound to a cornerstone of precision oncology.

## 12. Material and Methods

### 12.1. Literature Search Strategy

A comprehensive and systematic literature search was performed to identify and collate relevant studies investigating the molecular mechanisms underlying the anti-cancer effects of OC. The search strategy was conducted in accordance with the PRISMA guidelines to ensure methodological transparency. PubMed (National Library of Medicine, Bethesda, MD, USA) was employed as the primary database due to its extensive coverage of biomedical and life science literature. The search period was restricted from January 2005 to January 2025, based on the landmark discovery of OC’s chemical structure and biological activity by Beauchamp et al. in 2005 [32], which provided the foundational basis for subsequent mechanistic studies on OC.

A combination of Medical Subject Headings (MeSH) terms and free-text keywords were used in various Boolean combinations to maximize the retrieval of relevant publications. The search terms included but were not limited to the following: “Oleocanthal”, “Mediterranean Diet”, “Cancer”, “Tumor Suppression”, “Molecular Mechanisms”, “Apoptosis”, “Protease-Activated Receptor-2”, “Lysosomal Membrane Permeabilization”, “c-Met”, “STAT3”, “Epigenetic Modulation”, “Drug Resistance”, and “Metastasis”. Search filters were applied to restrict the retrieval to English-language, peer-reviewed, original research articles, clinical studies, and high-quality reviews containing primary experimental data. To ensure exhaustive coverage, additional hand-searching was conducted using the citation tracking of key articles and cross-referencing in Google Scholar and Scopus databases.

To contextualize the translational potential of OC as part of dietary interventions, a parallel search was performed on the ClinicalTrials.gov registry to identify completed or ongoing clinical trials evaluating the impact of the MD on cancer-related outcomes. Search terms used included “Mediterranean Diet”, “Cancer”, “Nutraceutical”, “Polyphenol”, and “Biomarker”. No restrictions were applied with regard to cancer type, geographical location, or study design. This search aimed to bridge preclinical mechanistic evidence with potential clinical applications of MD, particularly emphasizing its high OC content.

### 12.2. Eligibility Criteria and Study Selection

All identified records were subjected to an eligibility assessment based on pre-defined inclusion and exclusion criteria. Studies were included if they fulfilled the following conditions: (i) original experimental research (in vitro, in vivo, or ex vivo) evaluating the molecular mechanisms of OC in the context of cancer biology; (ii) studies elucidating the impact of OC on cellular signaling pathways, gene expression, apoptosis, metastasis, or drug resistance; and (iii) clinical trials and dietary intervention studies reporting cancer-related outcomes with clear association to the MD, where OC was recognized as a bioactive constituent.

Exclusion criteria encompassed the following: (i) review articles without primary data, commentaries, editorials, conference abstracts, and letters; (ii) studies focusing on olive oil phenolics other than OC without mechanistic evaluation of OC itself; and (iii) publications not available in English.

Two independent reviewers (SJ and YB; lead authors in this study) screened all titles and abstracts retrieved from the database search. Full-text articles were then assessed to ensure compliance with the inclusion criteria. Any discrepancies in study selection were resolved by discussion and consensus.

### 12.3. Data Extraction and Synthesis

A standardized data extraction form was developed to systematically collect relevant information from the eligible studies. Extracted variables included the following: (i) publication details (first author, year, journal); (ii) experimental design (cell lines used, animal models, human studies); (iii) sample size; (iv) cancer type and molecular targets investigated; (v) methodological platforms employed (e.g., Western blotting, RT-PCR, flow cytometry, transcriptomics, molecular docking, molecular dynamics simulations); (vi) key mechanistic findings; and (vii) limitations and quality indicators of the study. Data extraction was conducted independently by both reviewers (SJ and YB; lead authors in this study), with cross-validation for accuracy and consistency. Any disagreements were discussed until a consensus was achieved.

Given the methodological and experimental heterogeneity across the included studies, a formal quantitative meta-analysis was not feasible. Instead, a qualitative, narrative synthesis approach was employed to thematically organize and summarize the molecular pathways and biological processes modulated by OC. Studies were categorized according to mechanistic domains such as lysosomal membrane permeabilization, STAT3 inhibition, PAR-2 downregulation, c-Met inhibition, epigenetic modulation, and drug–drug interaction potential.

### 12.4. Chemical Structure Visualization

The 2D chemical structures of OC, oleacein, oleuropein, and hydroxytyrosol were retrieved from the PubChem database “https://pubchem.ncbi.nlm.nih.gov (accessed on 24 May 2025)” using their respective Compound Identification Numbers (CIDs). Structural files were downloaded in SDF format to preserve stereochemical and functional group information. These files were then imported into MarvinSketch (ChemAxon, Budapest, Hungary; version 24.3.0), a cheminformatics drawing tool, where the structures were rendered, annotated, and exported as high-resolution images for inclusion in the manuscript. Care was taken to preserve bond geometries, stereochemistry, and functional group labeling to ensure accurate representation of the molecular scaffolds.

### 12.5. Ethical Considerations

This review exclusively utilized secondary data extracted from publicly accessible, peer-reviewed publications and clinical trial registries. Therefore, institutional ethical approval was not required. The study adhered to established ethical standards for systematic literature reviews and complied with PRISMA guidelines.

### 12.6. Limitations

This review is subject to several inherent limitations. First, the search was confined to English-language publications, which may introduce language bias and limit the inclusion of potentially relevant studies published in other languages. Second, the heterogeneity of experimental models, dosing strategies, and outcome measures among the included studies precluded the execution of a formal meta-analysis. Third, publication bias cannot be excluded, as studies reporting negative or inconclusive results may be underrepresented in the published literature. Lastly, although relevant clinical trials assessing the MD were identified, the lack of trials specifically evaluating OC as an isolated intervention limits the translational extrapolation of preclinical findings to clinical practice.

## 13. Conclusions

In summary, OC emerges as a multifunctional, mechanistically versatile nutraceutical with significant promise in precision oncology. Through its pleiotropic modulation of cancer hallmarks—including lysosomal membrane permeabilization, suppression of oncogenic signaling cascades (c-MET/STAT3, PAR-2, COX-2/mPGES-1), inhibition of EMT and angiogenesis, and epigenetic remodeling—OC exerts selective cytotoxicity towards malignant cells while sparing non-cancerous tissues. Its ability to recalibrate tumor-promoting immune phenotypes, restore calcium homeostasis, and reprogram the tumor microenvironment underscores its potential not only as a cytotoxic agent but also as an immune and metabolic modulator.

However, OC’s clinical translation has been hindered by significant pharmacokinetic limitations, including poor oral bioavailability, rapid metabolism, and inadequate tumor targeting. Recent advances in delivery strategies—ranging from liposomal encapsulation and polymer-based nanoparticles to exosome-mediated delivery, microneedle-assisted transdermal systems, and peptide–drug conjugates—have shown promise in circumventing these limitations, enhancing OC’s stability, tissue specificity, and mechanistic precision. Furthermore, emerging data on OC’s bidirectional modulation of the gut microbiota, exemplified by suppression of oncogenic Bacteroides fragilis and enrichment of anti-inflammatory *Monoglobus* spp., add an additional layer of complexity to its systemic anti-cancer effects, potentially bridging tumor metabolism and host microbiome interactions.

Critically, our comprehensive analysis consolidates the preclinical evidence supporting OC’s synergistic potential with established chemotherapeutic, targeted, and immunotherapeutic agents across diverse malignancies—including prostate, pancreatic, colorectal, breast, and hematological cancers. By destabilizing key resistance mechanisms (e.g., AR-V7 splice variants, STAT3 activation, NLRP3 inflammasome assembly, and Bcl-2/Mcl-1-mediated apoptosis resistance), OC potentiates the efficacy of first-line and salvage therapies while simultaneously mitigating off-target toxicities.

Future research efforts must now focus on systematic pharmacokinetic profiling, validation in robust preclinical models—including organoid and patient-derived xenograft systems—and the development of GMP-grade delivery formulations optimized for human trials. Moreover, the elucidation of OC’s pharmacogenomic interactions and its impact on transporter-mediated drug efflux warrants further investigation to fully leverage its chemosensitizing capabilities.

Taken together, the converging lines of molecular, pharmacological, and translational evidence presented in this review firmly establish OC as a next-generation, dietary-derived anti-cancer candidate. Its integration into precision oncology frameworks, through rational combination regimens and advanced delivery technologies, holds considerable potential to augment therapeutic outcomes, particularly in inflammation-driven, drug-resistant, and metabolically reprogrammed malignancies.

## Figures and Tables

**Table 1:** A summary of the selected studies on effect of Mediterranean Diet.

Design	Region	Study Population	Methodology and Intervention	Key Results	References
Breast cancer					
Prospective study	Greece	14,807 women were followed up for an average of 9.8 years and 240 incident breast cancer cases were identified	Diet was assessed through a validated food frequency questionnaire, and conformity to the MD was evaluated through a score (range = 0–9 points) incorporating the characteristics of this diet.	Conformity to the traditional MD may be associated with lower breast cancer risk among postmenopausal women.	[38]
Case–control study	Spain	291 incident cases with confirmed breast cancer and 464 controls	A semi-quantitative food frequency questionnaire was completed; intake of monounsaturated fat and olive oil were measured.	The highest intake of monounsaturated fat was significantly related to a lower risk of breast cancer.	[39]
Prospective study	Northern Italy	8984 women were followed up for an average of 9.5 years and 207 incident breast cancer cases were identified	A semiquantitative food frequency questionnaire was used for the evaluation of four dietary patterns. Olive oil consumption was assessed in the salad vegetables pattern.	Findings suggest that a diet rich in raw vegetables and olive oil protects against breast cancer.	[40]
1:1:1 randomized, single-blind, controlled field trial	Spain	4282 women at high cardiovascular disease risk recruited after invitation by their primary care physicians to evaluate the effect of intervention on breast cancer incidence.	Participants were randomly assigned to one of three groups: a MD with extra-virgin olive oil, a MD with mixed nuts, or a control diet (focused on reducing fat intake). Adherence to the intervention diets was evaluated using a 14-item dietary screening questionnaire, while a 9-item questionnaire assessed adherence to the control diet.	After a median follow-up of 4.8 years, results suggest a beneficial effect of a MD supplemented with extra-virgin olive oil in the primary prevention of breast cancer.	[41]
Case–control study	Southern France	437 histological confirmed patients and 922 controls	Olive oil intake was assessed through a validated food frequency questionnaire.	A nonsignificant decreased association with cooked vegetables intake was observed.	[42]
Colorectal cancer					
Multicenter case–control study	Italy and Switzerland	1394 cases of colon cancer, 886 cases of rectal cancer and 4765 controls	Use of fried olive oil was measured.	Results do not indicate a relevant role of fried foods on CRC risk.	[43]
Prostate cancer					
Population-based case–control study	New Zealand	317 prostate cancer cases and 480 controls	Quantiles of monounsaturated fat-rich vegetable oil consumption were measured.	Increasing levels of MUFA-rich vegetable oil intake were associated with a progressive reduction in prostate cancer risk.	[44]
Population-based case–control study	Australia	858 men histological confirmed cancer and 905 age-frequency-matched men, selected at random from the electoral rolls	Various olive oil intakes were measured. A food frequency questionnaire with 121-items was used.	Some fatty acids (palmitoleic acid, 17:1 fatty acid, and 20:5 n-6) show weak inverse associations with prostate cancer risk.	[45]
Cancer of the larynx					
Combined dataset from two case–control studies	Italy and Switzerland	68 women histological confirmed cancer of the larynx. Controls were 340 women, admitted to the same network of hospitals	Intake of olive oil was measured. Validated food frequency questionnaire was used.	A vegetable-, fruit-, and olive oil-rich diet shows a protective effect, with lower odds ratios (OR = 0.3–0.5) observed among high consumers.	[46]
Case–control study	Italy and the Swiss Canton of Vaud	527 histological confirmed cases and 1297 frequency-matched controls	Dietary intakes 2 years prior to cancer diagnosis were estimated through a food frequency questionnaire. Olive oil consumption was measured.	Olive oil and some seed oils were linked to reduced laryngeal cancer risk (OR = 0.4–0.6), while mixed seed oils increased risk (OR = 2.2). The findings support a Mediterranean-style, plant-based diet with olive oil as a primary fat to lower cancer risk.	[47]
Cancer of the oral cavity and pharynx					
Multicenter case–control study in 14 centers	Multiple EU countries	1861 men and 443 women histological confirmed cancer patients and 1661 men and 566 women controls	Olive oil consumption was recorded through a semi-quantitative food frequency questionnaire, specifically developed for ARCAGE.	A diet rich in fruits, vegetables, olive oil, and tea is linked to a reduced risk of UADT cancers, suggesting a protective role (OR = 0.68–0.83).	[48]
Hospital-based case–control study	Greece	106 patients and 106 control subjects.	Different intakes of added lipids (olive oil is a substantial fraction) were measured. Dietary intake was assessed through a validated, semi-quantitative food frequency questionnaire.	Consumption of cereals, fruits, dairy products, and added lipids (which in Greece are represented mostly by olive oil) was found to be associated inversely with the risk of oral carcinoma.	[49]
Cancer of the esophagus					
Multicenter case–control study	Italy	304 patients and 743 controls	Olive oil intake was measured through a food frequency questionnaire which included 78 specific foods and beverages.	Among added lipids, olive oil intake showed a significant reduction in esophageal cancer risk, even after allowance for total vegetable consumption (OR = 0.4), while butter consumption was directly associated with this risk (OR = 2.2).	[50]
Stomach cancer					
Population-based case–control study	Italy	126 patients with MSI status (MSI+ = 43, MSI− = 83) and 561 controls	Tertiles of olive oil consumption and other lipids were measured.	For MSI− tumors, a protective effect was associated with frequent consumption of olive oil and with high intake of beta-carotene and other antioxidants.	[51]
Lung cancer					
Hospital-based case–control study	Italy	Cases were 342 patients with newly diagnosed primary lung cancer and controls were 292 adults	Olive oil intake was measured.	Exclusive use of olive oil and regular consumption of sage were significantly associated with reduced lung cancer risk, even after multivariate adjustment (OR = 0.67 and 0.43, respectively).	[52]
Ovarian cancer					
Multicenter case–control study	Italy	1031 histological confirmed patients and 2411 hospital controls	Seasonal lipid consumption, including olive oil and other fats, was measured using a food frequency questionnaire covering 78 specific foods and beverages.	The present study suggests a favorable effect of olive oil and other vegetable oils on ovarian cancer in this Italian population.	[53]
Endometrial cancer					
Hospital-based case–control study	Greece	84 histological confirmed patients and 84 controls with intact uterus	Olive oil was measured.	Data highly suggestive of protective effect of added lipids, which in the Greek diet are primarily represented by olive oil.	[54]
Bladder cancer					
Case–control study	Belgium	200 cases and 386 controls	Tertiles of olive oil intake were measured.	A protective effect of olive oil was concluded.	[55]

**Table 2:** OC content and stability across olive cultivars and processing methods (adapted from González-Rodríguez et al. [30]).

Olive Cultivar/Processing Method	OC Content/Stability
Coratina Cultivar	High OC content (~78.2 µg/mL)
Taggiasca Cultivar	Low OC content (~8.3 µg/mL)
Italian EVOO (general)	Up to 191.8 µg/mL OC
US EVOO (general)	Lower levels (~22.6 µg/mL)
Two-phase centrifugation	Higher OC retention

**Table 3:** Potential drug interactions affecting OC metabolism: CYP enzymes, mechanisms, and clinical relevance.

Drug Name	Drug Class/Indication	Enzyme Affected	Type of Interaction	Impact on OC Metabolism	Clinical Relevance
Imatinib	Tyrosine kinase inhibitor (CML)	CYP3A4	Inhibition	Reduces OC hydroxylation → ↑ OC plasma levels	Possible enhanced bioactivity/toxicity of OC
Tamoxifen	Estrogen receptor modulator (breast cancer)	CYP3A4, CYP2C9	Inhibition	Slows OC metabolism → ↑ OC systemic availability	May alter OC’s pharmacodynamic profile
Voriconazole	Antifungal (used in neutropenic cancer patients)	CYP3A4	Strong Inhibition	Impaired Phase I metabolism of OC	Risk of OC accumulation
Valproic Acid	Antiepileptic (chemotherapy-induced seizures)	UGT1A9, UGT2B7	Inhibition	Reduces OC glucuronidation → ↓ clearance	Potential OC accumulation, altered efficacy
Irinotecan	Topoisomerase inhibitor (colorectal cancer)	UGT1A1	Competitive Inhibition	Competes for UGT enzymes → Reduced OC conjugation	Potential pharmacokinetic variability of OC
Dexamethasone	Corticosteroid (antiemetic, edema in cancer)	CYP3A4	Induction	Enhances CYP3A4 activity → ↑ OC metabolism → ↓ plasma levels	Reduced OC therapeutic efficacy
Carbamazepine	Antiepileptic (chemotherapy-induced seizures)	CYP3A4, UGTs	Induction	Accelerates OC metabolism → ↓ bioavailability	Possible attenuation of OC’s beneficial effects
Phenytoin	Antiepileptic (chemotherapy-induced seizures)	CYP3A4, UGTs	Induction	Increases OC clearance	May necessitate higher dietary intake or formulation adjustments

“→” indicates progression or leads to the next step/outcome; “↑” denotes upregulation or an increase; “↓” denotes downregulation or a decrease.

**Table 4:** Detailed pharmacokinetic profile of OC and other notable polyphenols.

Polyphenol	Lipophilicity	Water Solubility	Bioavailability	Half-Life	Absorption	Metabolism	Systemic Circulation	Specific Remark (Cancer Focus)
Oleocanthal	High	Poor	Low (~5–15%) due to rapid metabolism	~1–2 h	Passive diffusion, enhanced by dietary fats	Extensive phase I and II metabolism (glucuronidation, sulfation)	Short half-life (~1–2 h), low plasma retention	Promising anti-cancer effects, but requires lipid-based formulations for improved tumor bioavailability.
Resveratrol	Moderate	Poor	Very low (~1–5%) due to extensive first-pass metabolism	~1–2 h	Passive diffusion, subject to efflux	Rapid glucuronidation and sulfation; high first-pass effect	Short half-life (~1–2 h), rapidly cleared	Known for anti-cancer properties but suffers from poor systemic availability; nano-formulations improve retention.
Curcumin	High	Extremely Poor	Extremely low (<1%) due to poor absorption and rapid metabolism	<1 h	Poor passive absorption, requires enhancers (e.g., piperine)	Primarily glucuronidation and sulfation, rapidly cleared	Very short half-life (<1 h), rapidly degraded	Strong anti-inflammatory and anti-cancer potential, but poor bioavailability limits therapeutic use without formulation aids.
EGCG	Low	High	Moderate (~5–10%) but affected by pH and enzymatic degradation	~2–4 h	Carrier-mediated transport, affected by pH	Methylation, glucuronidation, sulfation; oxidative degradation	Moderate (~2–4 h); better than curcumin but still transient	Anti-cancer properties via apoptosis induction; limited by pH sensitivity and enzymatic breakdown.
Quercetin	Moderate	Poor	Moderate to high (~20–50%) due to enterohepatic recirculation	~3–12 h	Active transport via sodium-dependent transporters	Glucuronidation and sulfation, but slow metabolism allows higher circulation	Long half-life (~3–12 h); prolonged circulation due to conjugation	Best systemic retention; exhibits strong anti-cancer properties by modulating multiple oncogenic pathways.

**Table 5:** Delivery strategies for OC: encapsulation methods, release kinetics, and tumor targeting efficiency.

Delivery System	Encapsulation Efficiency	Release Kinetics	Tumor Targeting Metrics
Liposomes	60–80% [96]	Slow (≤40% in 24 h), Fickian diffusion [96]	Passive targeting via EPR effect
Exosomes	Variable, typically ≤50% [97]	Sustained, natural endocytosis-mediated release [97]	Inherent homing capacity, BBB penetration [98]
Microneedles	High (>80%) due to nanoparticle integration [99]	Localized burst release in acidic TME [99]	Direct tumor microenvironment penetration [100]
Metal–Organic Frameworks	~50% *w*/*w* for ZIF-8 [101]	Stimuli-responsive (e.g., pH/glucose-sensitive) [101]	Ligand-targeted, e.g., folate/glucose-conjugated [101]
Dendrimer-Based Nanocarriers	High (>85%) for hydrophobic drugs [102]	Controlled intracellular release, enzyme-sensitive [102]	Surface modification with ligands (e.g., GalNAc) [102]

**Table 6:** Anti-cancer synergistic potential of OC: mechanisms, experimental models, and potential outcomes by cancer type.

Cancer Type	OC Mechanism	Synergistic Therapy	Experimental Models	Potential Outcome
Prostate Cancer	Inhibits SMYD2-mediated AR methylation, destabilizing AR and AR-V7; sensitizes to AR antagonists, PARP inhibitors, and taxanes.	Enzalutamide, Apalutamide, Olaparib (PARP inhibitors), Docetaxel	LNCaP/AR+ xenografts; AR degradation and PSA monitoring	Delays AR antagonist resistance; sensitizes to PARP inhibitors; enhances docetaxel efficacy.
Pancreatic Ductal Adenocarcinoma	Suppresses EMT via Snail/Slug-Twist downregulation; inhibits autophagy; counteracts bacterial infection-driven metastasis.	FOLFIRINOX, Gemcitabine, Gentamicin, MEK inhibitors (Trametinib)	KRASG12D organoids; orthotopic models with LPS challenge	Inhibits EMT; reduces autophagy-mediated resistance; suppresses infection-driven metastasis.
Glioblastoma Multiforme	Reduces NF-κB and COX-2; downregulates MGMT via TNF-α inhibition; potential BBB penetration.	Temozolomide (TMZ)	Brain organoids with glioblastoma stem cells; IDH1-mutant organoids	Sensitizes to TMZ by reducing MGMT expression; disrupts stem cell niche.
Multiple Myeloma	Disrupts MIP-1α/CCL3 signaling; downregulates Bcl-2; increases proteotoxic stress; enhances mitochondrial priming.	Venetoclax, Carfilzomib	MM-HS5 stromal cell co-cultures; 3D bone marrow scaffolds	Enhances apoptosis; increases mitochondrial priming; overcomes proteasome inhibitor resistance.
Acute Myeloid Leukemia	Suppresses STAT3 in FLT3-ITD mutant cells; generates ROS; enhances effect of FLT3 and topoisomerase inhibitors.	Gilteritinib (FLT3 inhibitor), Etoposide	AML patient-derived xenografts (PDX); TP53 mutant models	Amplifies chemotherapy-induced DNA damage; suppresses STAT3 survival signals.

## Data Availability

The datasets generated and/or analyzed during the current study are not publicly available but are accessible from the corresponding author upon reasonable request. Interested researchers may contact the corresponding author for data access inquiries, subject to compliance with any applicable privacy or confidentiality obligations.

## Abbreviations

COX	Cyclooxygenase
NSCLC	Non-Small Cell Lung Cancer
EGFR	Epidermal Growth Factor Receptor
MAPK	Mitogen-Activated Protein Kinase
MET	Mesenchymal–Epithelial Transition factor (a receptor tyrosine kinase)
TNF-α	Tumor Necrosis Factor-alpha
IL-6	Interleukin-6
NF-κB	Nuclear Factor-kappa B
STAT3	Signal Transducer and Activator of Transcription 3
EMT	Epithelial–Mesenchymal Transition
CAFs	Cancer-Associated Fibroblasts
PAR-2	Protease-Activated Receptor 2
CRC	Colorectal Cancer
EVOO	Extra Virgin Olive Oil
OC	Oleocanthal
LMP	Lysosomal Membrane Permeabilization
HGF	Hepatocyte Growth Factor
PI3K/AKT	Phosphoinositide 3-Kinase/Protein Kinase B
RAS/ERK	Rat Sarcoma/Extracellular Signal-Regulated Kinase
NLRP3	NOD-like Receptor Family Pyrin Domain Containing 3
PGE2	Prostaglandin E2
mPGES-1	Microsomal Prostaglandin E Synthase-1
VEGF	Vascular Endothelial Growth Factor
VEGFR2	Vascular Endothelial Growth Factor Receptor 2
TSP-1	Thrombospondin-1
TME	Tumor Microenvironment
MMP	Matrix Metalloproteinase
ROS	Reactive Oxygen Species
GSH	Glutathione
PPP	Pentose Phosphate Pathway
TCA	Tricarboxylic Acid Cycle (Krebs Cycle)
OXPHOS	Oxidative Phosphorylation
ETC	Electron Transport Chain
FAO	Fatty Acid Oxidation
FASN	Fatty Acid Synthase
CPT1A	Carnitine Palmitoyltransferase 1A
SREBP-1	Sterol Regulatory Element-Binding Protein 1
IOM	Immuno-Oncology-Microbiome
SCFA	Short-Chain Fatty Acids
TPs	Tea Polyphenols
BRCA1/2	Breast Cancer Gene 1/2
MMR	Mismatch Repair
FAP	Familial Adenomatous Polyposis
IBD	Inflammatory Bowel Disease
PDAC	Pancreatic Ductal Adenocarcinoma
TMZ	Temozolomide
MGMT	O6-Methylguanine-DNA Methyltransferase
MM	Multiple Myeloma
AML	Acute Myeloid Leukemia
FLT3-ITD	FMS-like Tyrosine Kinase 3-Internal Tandem Duplication
AR	Androgen Receptor
SMYD2	SET and MYND Domain-Containing Protein 2
PARP	Poly (ADP-Ribose) Polymerase
CHS	Chondrosarcoma
IDH1/2	Isocitrate Dehydrogenase 1/2
2-HG	2-Hydroxyglutarate
COL2A1	Collagen Type II Alpha 1 Chain
TAMs	Tumor-Associated Macrophages
LPHNs	Lipid–Polymer Hybrid Nanoparticles
SOCE	Store-Operated Calcium Entry
MSS	Microsatellite Stable
MSI-H	Microsatellite Instability-High
PDX	Patient-Derived Xenograft
CMS4	Consensus Molecular Subtype 4 (Colorectal Cancer)
PK-PD	Pharmacokinetic–Pharmacodynamic
GMP	Good Manufacturing Practice
LC-MS/MS	Liquid Chromatography-Tandem Mass Spectrometry
DEPs	Differentially Expressed Proteins
PTM	Post-Translational Modification
SRM/PRM	Selected/Parallel Reaction Monitoring
ChIP-seq	Chromatin Immunoprecipitation Sequencing
CRISPR	Clustered Regularly Interspaced Short Palindromic Repeats
scRNA-seq	Single-Cell RNA Sequencing
AI	Artificial Intelligence
CNN	Convolutional Neural Network
GNN	Graph Neural Network
VAE	Variational Autoencoder
PINN	Physics-Informed Neural Network
QM/MM	Quantum Mechanics/Molecular Mechanics
SHAP	SHapley Additive exPlanations
Mol D	Molecular Dynamics
RMSD	Root Mean Square Deviation
RMSF	Root Mean Square Fluctuation
MM/PBSA	Molecular Mechanics/Poisson-Boltzmann Surface Area
FEP	Free Energy Perturbation
SPR	Surface Plasmon Resonance
MST	Microscale Thermophoresis
BLI	Biolayer Interferometry
